# Efficacy and safety of disease-modifying oral drugs in treatment of relapsing-remitting multiple sclerosis: systematic review and network meta-analysis

**DOI:** 10.3389/fimmu.2026.1733948

**Published:** 2026-03-16

**Authors:** Yonghong Zhao, Bokun Chen, Xiaojuan Zhao, Ruimeng Yang, Wenjuan He, Dan Li, Qian Sun, Jiaxi Zhang, Xiuju Liu

**Affiliations:** Department of Pharmacy, The Second Hospital of Hebei Medical University, Shijiazhuang, China

**Keywords:** acceptability, efficacy, network meta-analysis, oral drugs, randomized controlled trial, relapsing-remitting multiple sclerosis

## Abstract

**Background:**

Relapsing-Remitting Multiple Sclerosis (RRMS) is a chronic inflammatory demyelinating disease affecting the central nervous system, characterized by complex pathogenesis and increasing annual incidence rates. Although current clinical interventions for RRMS are diverse, there remains a relative scarcity of direct comparative studies on the efficacy and safety profiles among different oral disease-modifying drugs, resulting in insufficient comprehensive evidence.

**Objective:**

This study aims to apply network meta-analysis techniques to systematically evaluate the relative efficacy and safety of different disease-modifying oral drugs in the treatment of RRMS, clarify their differences, and provide high-quality evidence-based medical support for optimal clinical treatment decision-making.

**Methods:**

The systematic search was conducted in PubMed, Embase, Web of Science, and The Cochrane Register of Clinical Trials databases, covering the period from database inception to July 31, 2025. Randomized controlled trials were included, with study populations consisting of adult patients with relapsing-remitting multiple sclerosis, interventions involving disease-modifying oral medications, and comparators being placebo or other treatments. The primary data sources were phase II/III clinical trials, with non-standard treatment regimens serving as crucial observational approaches and thus analyzed as independent nodes for comparison. Primary outcome measures included Annualized relapse rate and Adverse events leading to discontinuation, while secondary outcomes comprised Adverse events, Serious adverse events, active T1 lesions, and active T2 lesions. A random-effects model was employed for network meta-analysis, with dichotomous and continuous variables analyzed using odds ratios (OR) and mean differences (MD) along with their respective 95% confidence intervals (CI) as effect measures to compare various interventions. Treatment rankings were performed using the surface under the cumulative ranking curve (SUCRA) probability method.

**Results:**

A total of 15 RCTs involving 14,869 participants were included. The NMA results demonstrated that Siponimod, Ponesimod, Laquinimod, Fingolimod, Cladribine, and Dimethyl Fumarate were all superior to placebo in reducing annualized relapse rates in patients with multiple sclerosis, with Siponimod (2 mg; SUCRA = 86.9%) and Laquinimod 0.3 mg (SUCRA = 10.1%) being the best and worst treatments, respectively. Regarding adverse events leading to study discontinuation, the optimal and least favorable interventions were Fingolimod (0.25 mg; SUCRA = 83.1%) and Siponimod (10 mg; SUCRA = 3.1%), respectively.

**Conclusions:**

This NMA demonstrated that Siponimod (2mg) is the most efficacious therapeutic intervention; FIN (0.25 mg) exhibited relative safety advantages in terms of DAE, though this dosage constitutes an exploratory regimen and should not serve as the basis for routine clinical medication. However, these findings still require further validation in subsequent studies.

**Systematic Review Registration:**

https://www.crd.york.ac.uk/prospero/, identifier CRD420250654500.

## 1 Introduction

Multiple sclerosis (MS) is a chronic, inflammatory, demyelinating, and neurodegenerative disease of the central nervous system (CNS) (1). Affecting 2.8 million people worldwide annually, MS is also considered a major contributor to the global economic health burden (2). Both genetic and environmental factors play significant roles in the pathogenesis of MS by activating immune responses and inducing inflammation. MS patients may exhibit diverse clinical courses, with approximately 80-85% initially presenting with relapsing-remitting MS (RRMS) at first diagnosis (3). RRMS is characterized by recurrent acute exacerbations of neurological dysfunction (relapses), followed by varying degrees of remission after acute episodes. However, as the disease progresses, patients often experience gradual accumulation of neurological impairments with limited recovery (4).

Currently, Disease-Modifying Therapies (DMTs) have become the core strategy for treating RRMS, aiming to reduce relapse rates, delay disability progression, and preserve neurological function as much as possible (5). Among various DMTs, oral disease-modifying drugs have significantly improved treatment adherence due to their convenience, offering new hope for RRMS patients (6). The currently available oral DMTs exhibit distinct mechanisms of action, which can be classified as follows: pyrimidine synthesis inhibitors (e.g., Teriflunomide [TER]) mitigate central nervous system inflammation by suppressing the proliferation of activated T and B lymphocytes while preserving protective immune responses (7); S1P receptor modulators (e.g., Fingolimod [FIN], Ponesimod [PON], Ozanimod [OZA], Siponimod [SIP]) sequester lymphocytes in lymph nodes, preventing their migration to the CNS and thereby achieving reversible immunomodulation (8, 9); Nrf2 activators (e.g., Dimethyl Fumarate [DMF], Diroximel Fumarate [DRF]) enhance antioxidant capacity in CNS cells through Nrf2 pathway activation, exerting neuroprotective effects (10); purine analogs (e.g., Cladribine [2-CdA]) induce lymphocyte apoptosis to achieve immune reconstitution (11); and quinoline-3-carboxamide multitarget immunomodulators (e.g., Laquinimod [LAQ]) combine both immunomodulatory and neuroprotective effects (12–14). These oral DMTs offer greater convenience compared to intravenous administration. Their diverse mechanisms, such as inhibiting immune cell trafficking or depleting immune cells, facilitate better patient adaptation to treatment, thereby significantly expanding therapeutic options (15–17). However, there remains a paucity of head-to-head comparative studies among different oral DMTs, with most existing evidence derived from placebo-controlled trials. Direct comparative data on efficacy and safety between oral DMTs are notably lacking. Therefore, we conducted a network meta-analysis to compare the effectiveness and safety of approved oral DMTs in RRMS patients, aiming to provide evidence-based guidance for clinical decision-making.

## 2 Methods

This study has been registered on the international prospective systematic review registration platform PROSPERO (URL: https://www.crd.york.ac.uk/prospero/), with registration number CRD420250654500. The research content was prepared in accordance with the Preferred Reporting Items for Systematic Reviews and Meta-Analyses (PRISMA) guidelines. The software tools involved in this study include EndNote X8 (for literature management and manuscript writing), Excel 2019 (for data extraction and organization), Review Manager 5.3 (for methodological quality assessment, conventional meta-analysis, and heterogeneity testing), and Stata 17.0 (for network meta-analysis (NMA), inconsistency testing, SUCRA plots, rankograms, and funnel plots).

### 2.1 Inclusion and exclusion criteria and search strategy

Inclusion and exclusion criteria were established based on the PICOS principle (**Table 1**).

**Table 1:** Inclusion and exclusion criteria.

PICOS	Inclusion	Exclusion
Population(P)	Adult patients with RRMS	
Intervention(I)	Teriflunomide、Fingolimod、Ozanimod、Ponesimod、Laquinimod,、Siponimod、Dimethyl Fumarate、Diroximel fumarate、Monomethyl fumarate、Cladribine	
Comparison(C)	Other marketed medications for treating relapsing-remitting multiple sclerosis or placebo.	
Outcome(O)	Primary efficacy endpoint:annualized relapse rate(ARR) Adverse events leading to discontinuation(DAE); Secondary efficacy endpoints:magnetic resonance imaging(MRI):active T1 lesions, and active T2 lesions Adverse events(AE) serious adverse event(SAE)	
Study design(S)	randomized controlled trial(RCT)	Open-label trial Clinical trials with unpublished data Research that cannot be obtained or duplicated for publication.

Computerized searches were conducted in the PubMed, Embase, Web of Science, and The Cochrane Register of Clinical Trials databases. The search period spanned from database inception to July 31, 2025. A combination of subject headings and free-text terms was employed for the search strategy.Taking PubMed as an example, the specific search strategy is as follows:

(“Teriflunomide”[Supplementary Concept] OR “Teriflunomide”[Title/Abstract] OR (“Aubagio”[Title/Abstract] OR “HMR1726”[Title/Abstract]) OR “Fingolimod Hydrochloride”[MeSH Terms] OR (“Fingolimod”[Title/Abstract] OR “Gilenya”[Title/Abstract] OR “Gilenia”[Title/Abstract] OR “FTY720”[Title/Abstract]) OR “Amiselimod”[Supplementary Concept] OR (“Amiselimod”[Title/Abstract] OR “MT-1303”[Title/Abstract]) OR “Ozanimod”[Supplementary Concept] OR (“Ozanimod”[Title/Abstract] OR “Zeposia”[Title/Abstract] OR “RPC1063”[Title/Abstract]) OR “Ponesimod”[Supplementary Concept] OR (“Ponesimod”[Title/Abstract] OR “ponvory”[Title/Abstract] OR “ACT128800”[Title/Abstract]) OR “Laquinimod”[Supplementary Concept] OR (“Laquinimod”[Title/Abstract] OR “ABR215062”[Title/Abstract]) OR “Siponimod”[Supplementary Concept] OR (“Siponimod”[Title/Abstract] OR “Mayzent”[Title/Abstract] OR “BAF312”[Title/Abstract]) OR (“Ceralifimod”[Supplementary Concept] OR “Dimethyl Fumarate”[MeSH Terms] OR “Monomethyl fumarate”[Supplementary Concept] OR “Cladribine”[MeSH Terms] OR “Evobrutinib”[Supplementary Concept] OR “Fenebrutinib”[Supplementary Concept] OR “Sphingosine-1-Phosphate Receptors”[MeSH Terms] OR “Agammaglobulinaemia Tyrosine Kinase”[MeSH Terms]) OR (“Ceralifimod”[Title/Abstract] OR “ONO-4641”[Title/Abstract] OR “Dimethyl Fumarate”[Title/Abstract] OR “Tecfidera”[Title/Abstract] OR “FAG201”[Title/Abstract] OR “BG00012”[Title/Abstract] OR “BG-12”[Title/Abstract] OR “Fumaderm”[Title/Abstract] OR “DMF”[Title/Abstract] OR “Diroximel fumarate”[Title/Abstract] OR “Vumerity”[Title/Abstract] OR “DRF”[Title/Abstract] OR “Monomethyl fumarate”[Title/Abstract] OR “Bafiertam”[Title/Abstract] OR “Cladribine”[Title/Abstract] OR “Mavenclad”[Title/Abstract] OR “Orelabrutinib”[Title/Abstract] OR “Evobrutinib”[Title/Abstract] OR “Tolebrutinib”[Title/Abstract] OR “SAR442168”[Title/Abstract] OR “Fenebrutinib”[Title/Abstract] OR “GDC-0853”[Title/Abstract] OR “BIIB091”[Title/Abstract] OR “Sphingosine-1-phosphate”[Title/Abstract] OR “brutons tyrosine kinase”[Title/Abstract])) AND (“Multiple Sclerosis”[MeSH Terms] OR (“Multiple Sclerosis”[All Fields] OR (“ms”[Journal] OR “med sci paris”[Journal] OR “ms”[All Fields]))).

### 2.2 Literature screening and data extraction

The retrieved literature was exported to EndNote X8 software based on the aforementioned search strategy. The software’s automatic deduplication function was utilized to roughly remove duplicates by limiting titles, authors, and publication years. Following the inclusion and exclusion criteria, two researchers independently screened the titles and abstracts of the literature for preliminary selection, excluding those that did not meet the criteria and recording the reasons for exclusion. The remaining literature underwent full-text review for secondary screening, with further exclusions made based on the criteria and reasons documented. Subsequently, the two researchers cross-checked the included literature and jointly decided on final inclusion. Any disagreements were resolved through consultation with a third researcher. Excel 2019 was employed for literature management and data extraction, with key information extracted into a pre-designed data extraction form, including patient demographics, interventions, disease course, affected populations, and outcome measures. Additionally, for clinical trial information from ClinicalTrials.gov, a similar method was adopted for online screening and extraction. The included data primarily originated from Phase II/III clinical trials, with non-standard treatment regimens serving as crucial observational measures and thus analyzed as independent nodes for comparison.

### 2.3 Methodological quality assessment

The methodological quality of the included studies was independently evaluated by two researchers, with any disagreements resolved through discussion with a third researcher. The quality of the literature was assessed using the quality evaluation criteria provided by the Cochrane Collaboration, encompassing seven aspects: random sequence generation; allocation concealment; blinding of participants and personnel; blinding of outcome assessment; incomplete outcome data; selective reporting; and other biases. The evaluations were then categorized as “low risk,” “high risk,” or “unclear.”

### 2.4 Statistical analysis

#### 2.4.1 Pairwise meta-analysis

This study employed fixed-effects and random-effects models to conduct traditional pairwise meta-analysis. The outcome measures were categorized as dichotomous variables and continuous variables. For dichotomous variables (AEs, DAEs, and SAEs in this study), odds ratios (ORs) with 95% confidence intervals (CIs) were used as effect size statistics. For continuous variables (ARR, T1, and T2 in this study), mean differences (MDs) with 95% CIs were calculated to evaluate the pooled effect size. The magnitude of heterogeneity was assessed using the I² statistic, with 50% serving as the threshold for determining significant between-study heterogeneity. When I² < 50%, a fixed-effects model was applied; when I² ≥ 50%, a random-effects model was employed to account for heterogeneity effects.

#### 2.4.2 Network meta-analysis

In the frequency framework, a random-effects model NMA was conducted, with dichotomous and continuous outcomes expressed as OR and MD values, respectively, along with their respective 95% CIs. The global inconsistency model was used to assess the consistency of the entire network, with P > 0.05 indicating good consistency. The loop-specific approach was employed to evaluate the consistency of each closed loop in the NMA; if the information from two sources of evidence (direct and indirect comparisons) was sufficiently similar, they could be combined. The specific identification method involved calculating inconsistency factors (IF) and their 95% CIs to define the consistency of each closed loop, with a 95% CI lower limit including 0 considered satisfactory consistency. The side-splitting method was also used to assess the inconsistency of the model, with P > 0.05 for each node indicating good consistency and the possibility of combination. The Surface Under the Cumulative Ranking (SUCRA) was used to rank each intervention for each indicator, with a larger area under the curve indicating a higher rank and suggesting a superior intervention. A comparison-adjusted funnel plot was drawn to detect potential publication bias in small and large study results (typically requiring ≥10 studies). The principle involves calculating the difference between the effect size of a specific pairwise comparison in each study and the pooled effect size of all similar comparisons, then regressing the calculated difference based on the standard error (SE) of the effect size. This adds a simple linear regression line to the funnel plot, aiding in a more intuitive exploration of the bias in study results.

## 3 Results

### 3.1 Study selection and characteristics of included studies

**Figure 1:**
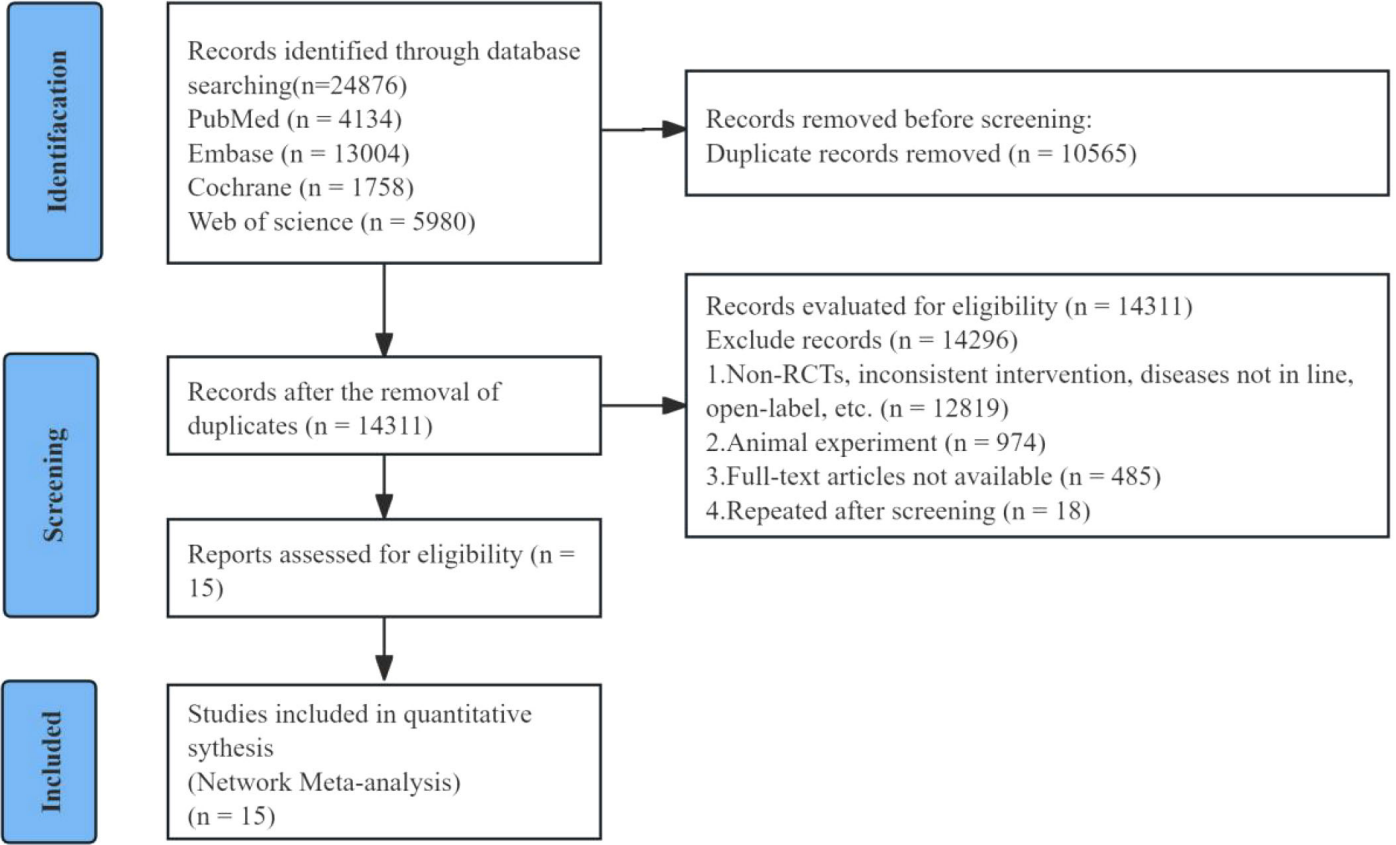
Flow diagram of literature search and study selection.

A total of 24,876 articles were retrieved, and ultimately 15 double-blind, parallel, randomized controlled trials (RCTs) conducted between 2008 and 2022 were included (2 two-arm studies, 10 three-arm studies, and 4 four-arm studies), all of which had been published. These studies compared 6 oral DMT drugs, 2 injectable DMT drugs, and placebo. The research involved a total of 14,869 participants (10,679 randomly assigned to the active drug group and 4,190 to the placebo group), with treatment durations ranging from 12 to 96 weeks. Among the 15 studies, the trial conducted by Selmaj K et al. in 2013 included two cohorts. The literature search and screening process is shown in **Figure 1**, and the basic characteristics of the studies are presented in **Table 2**.

**Table 2:** Characteristics of included studies.

References	NCT	Treating arm	Regimen	Number (female/male)	Age (years, means ± SD)	Baseline EDSS score(means ± SD or mean(min, max))	Treatment period (week)	Outcomes
Giovannoni G,2010 (18)	NCT00213135 phase 3 trial	Cladribine 5.25 mg/kg	cumulative dose 5.25 mg/kg, PO	456(312/144)	39.1 ± 9.9	3.0 ± 1.4	96	ARR AE SAE
		Cladribine 3.5 mg/kg	cumulative dose 3.5 mg/kg, PO*	433(298/135)	37.9 ± 10.3	2.8 ± 1.2	96	
		Placebo	Placebo, PO	437(288/149)	38.7 ± 9.9	2.9 ± 1.3	96	
Kappos L,2009 (19)	NCT00168701 phase 2 trial	Dimethyl Fumarate 120 mg	120 mg, PO, QD	64(42/22)	34.8 ± 10.2	2.52 ± 1.11	24	ARR T1 T2 AE DAE
		Dimethyl Fumarate 120 mg	120 mg, PO, TID	64(44/20)	36.3 ± 9.5	2.51 ± 1.02	24	
		Dimethyl Fumarate 240 mg	240 mg, PO, TID	63(42/21)	37.3 ± 9.1	2.87 ± 1.33	24	
		Placebo	Placebo, PO, TID	65(36/29)	35.6 ± 8.2	2.67 ± 1.23	24	
Fox RJ,2012 (20)	NCT00451451 phase 3 trial	Dimethyl Fumarate 240 mg	240 mg, PO,BID*	359(245/114)	37.8 ± 9.4	2.6 ± 1.2	96	ARR AE DAE SAE
		Dimethyl Fumarate 240 mg	240 mg, PO, TID	345(250/95)	37.8 ± 9.4	2.5 ± 1.2	96	
		Placebo	Placebo, PO, TID	363(251/112)	36.9 ± 9.2	2.6 ± 1.2	96	
		Glatiramer acetate 20 mg	20mg,SC,QD*	350(247/103)	36.7 ± 9.1	2.6 ± 1.2	96	
Gold R,2012 (5)	NCT00420212 phase 3 trial	Dimethyl Fumarate 240 mg	240 mg, PO, BID*	410(296/114)	38.1 ± 9.1	2.40 ± 1.29	48	ARR AE DAE SAE
		Dimethyl Fumarate 240 mg	240 mg, PO, TID	416(306/110)	38.8 ± 8.8	2.36 ± 1.19	48	
		Placebo	Placebo, PO, TID	408(306/102)	38.5 ± 9.1	2.48 ± 1.24	48	
Saida T,2019 (21)	NCT01838668 phase 3 trial	Dimethyl Fumarate 240 mg	240 mg, PO, BID*	111(70/41)	37.3 ± 8.3	2.2 ± 1.3	24	AE DAE SAE
		Placebo	Placebo, PO, BID	113(74/39)	36.0 ± 7.5	1.9 ± 1.3	24	
Cohen JA,2010 (22)	NCT00340834 phase 3 trial	Fingolimod 1.25 mg	1.25 mg, PO, QD	426(293/133)	35.8 ± 8.4	2.21 ± 1.31	48	ARR AE DAE SAE
		Fingolimod 0.5 mg	0.5 mg, PO, QD*	431(282/149)	36.7 ± 8.8	2.24 ± 1.33	48	
		Interferon β-1a 30 µg	30 µg, IM, QW*	435(295/140)	36.0 ± 8.3	2.19 ± 1.26	48	
Kappos L,2010 (23)	NCT00289978 phase 3 trial	Fingolimod 1.25 mg	1.25 mg, PO, QD	429(295/134)	37.4 ± 8.9	2.4 ± 1.4	96	ARR AE DAE SAE
		Fingolimod 0.5 mg	0.5 mg, PO, QD*	425(296/129)	36.6 ± 8.8	2.3 ± 1.3	96	
		Placebo	Placebo, PO, QD	418(298/120)	37.2 ± 8.6	2.5 ± 1.3	96	
Calabresi PA,2013 (24)	NCT00355134 phase 3 trial	Fingolimod 1.25 mg	1.25 mg, PO, QD	370(281/89)	40.9 ± 8.90	2.5 ± 1.3	96	ARR AE DAE SAE
		Fingolimod 0.5 mg	0.5 mg, PO, QD*	358(275/83)	40.6 ± 8.4	2.4 ± 1.3	96	
		Placebo	Placebo, PO, QD	355(288/67)	40.1 ± 8.4	2.4 ± 1.3	96	
Cree BAC,2020 (25)	NCT01633112 phase 3 trial	Fingolimod 0.5 mg	0.5 mg, PO, QD*	352(264/88)	40.3 ± 11.1	2.74 ± 1.46	48	ARR AE DAE SAE
		Fingolimod 0.25 mg	0.25 mg, PO, QD	370(276/94)	38.9 ± 11.0	2.55 ± 1.41	48	
		Glatiramer acetate 20 mg	20mg,SC,QD*	342(252/90)	39.6 ± 10.8	2.73 ± 1.42	48	
Comi G,2008 (26)	NCT00349193 phase 2 trial	Laquinimod 0.6 mg	0.6 mg, PO, QD*	106	/	2.3 ± 1.0	36	ARR T1 AE DAE SAE
		Laquinimod 0.3 mg	0.3 mg, PO, QD	98	/	2.3 ± 1.1	36	
		Placebo	Placebo, PO, QD	102	/	2.5 ± 1.1	36	
Comi G,2012 (27)	NCT00509145 phase 3 trial	Laquinimod 0.6 mg	0.6 mg, PO, QD*	550(391/159)	38.9 ± 9.2	2.6 ± 1.3	24	ARR AE DAE SAE
		Placebo	Placebo, PO, QD	556(368/188)	38.5 ± 9.1	2.6 ± 1.3	24	
T.L.Vollmer,2014 (28)	NCT00605215 phase 3 trial	Laquinimod 0.6 mg	0.6 mg, PO, QD*	434(282/152)	37.0 ± 9.3	2.5 (1.5, 3.5)	96	ARR AE DAE SAE
		Interferon β-1a 30 µg	30 µg, IM, QW*	447(307/140)	38.2 ± 9.5	2.5 (1.5, 3.5)	96	
		Placebo	Placebo, PO, QD	450(321/129)	37.5 ± 9.5	2.5 (1.5, 3.5)	96	
Comi G,2022 (29)	NCT01707992 phase 3 trial	Laquinimod 0.6 mg	0.6 mg, PO, QD*	727(510/217)	36.8 ± 9.3	2.6 ± 1.3	96	ARR AE DAE SAE
		Laquinimod 1.2 mg	1.2 mg, PO, QD	732(475/257)	36.1 ± 9.22	/	96	
		Placebo	Placebo, PO, QD	740(488/252)	35.9 ± 9.0	2.7 ± 1.2	96	
Olsson T,2014 (30)	NCT01006265 phase 2 trial	Ponesimod 10 mg	10 mg, PO, QD*	108(71/37)	36.9 ± 9.2	2.4 ± 1.25	24	ARR T1 T2 AE SAE
		Ponesimod 20 mg	20 mg, PO, QD*	114(77/37)	35.5 ± 8.5	2.2 ± 1.31	24	
		Ponesimod 40 mg	40 mg, PO, QD	119(79/40)	36.5 ± 8.5	2.2 ± 1.17	24	
		Placebo	Placebo, PO, QD	121(85/36)	36.6 ± 8.6	2.2 ± 1.23	24	
Selmaj K,2013 (31) cohort 1	NCT00879658 phase 2 trial	Siponimod 10 mg	10 mg, PO, QD	50(30/20)	36.4 ± 8.4	2·3 ± 1.0	24	ARR T1 T2 AE DAE SAE
		Siponimod 2 mg	2 mg, PO, QD*	49(34/15)	37.4 ± 8.9	2.4 ± 1.2	24	
		Siponimod 0.5 mg	0.5 mg, PO, QD*	43(30/13)	36.0 ± 8.8	2.2 ± 1.3	24	
		Placebo	Placebo, PO, QD	46(36/10)	35.2 ± 8.75	2.3 ± 1.1	24	
Selmaj K,2013 cohort 2		Siponimod 1.25 mg	1.25 mg, PO, QD*	42(31/11)	35.4 ± 8.9	2.0 ± 1.0	12	AE DAE SAE
		Siponimod 0.25 mg	0.25 mg, PO, QD*	51(42/9)	37.4 ± 8.4	2.3 ± 1.1	12	
		Placebo	Placebo, PO, QD	16(9/7)	35.9 ± 8.24	2.3 ± 1.1	12	

IM, intramuscular injection; SC, subcutaneous injection; PO, orally; QD, once daily; BID, twice daily; TID=three times daily; QW=weekly;*=Approved dosage for marketing.

### 3.2 Methodological quality assessment of included studies

**Figure 2:**
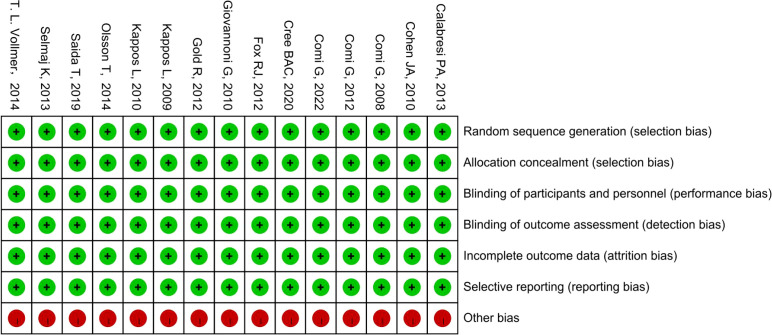
Risk of Bias Summary. Green circles indicate low risk, red circles indicate high risk, and yellow circles indicate unclear risk.

All RCTs reported the methods of random sequence generation, employing parallel-group or double-blind double-dummy allocation schemes with proper allocation concealment. Each study provided complete outcome data without selective reporting, and implemented blinding of investigators, participants, and outcome assessors. All studies received sponsor funding; however, as they were pre-marketing clinical trials with relatively high credibility, the funding did not significantly affect the risk of bias. Overall, the included studies demonstrated low risk of bias (**Figure 2**).

### 3.3 The efficacy and acceptability of the primary outcome measures

#### 3.3.1 Pairwise meta-analysis

In traditional meta-analyses, the I² statistic was employed to assess heterogeneity in direct comparisons. Regarding ARR, **Supplementary Figure 1A** incorporated 17 interventions for fixed-effects model meta-analysis, demonstrating I²≤6% across all direct comparisons with no significant heterogeneity. Interventions significantly superior to placebo included DMF 240mg BID [MD = -0.19, 95% CI (-0.25, -0.13)] and FIN 0.5mg [MD = -0.20, 95% CI (-0.25, -0.15)]. For DAE, **Supplementary Figure 1B** analyzed 14 interventions, revealing I²≤75% across all direct comparisons, thus necessitating random-effects model meta-analysis. Results indicated FIN 0.5mg and PBO significantly outperformed FIN 1.25mg, with effect sizes of [OR = 1.97, 95% CI (1.40, 2.76)] and [OR = 2.04, 95% CI (1.50, 2.78)] respectively.

#### 3.3.2 Evidence network diagram

**Figure 3:**
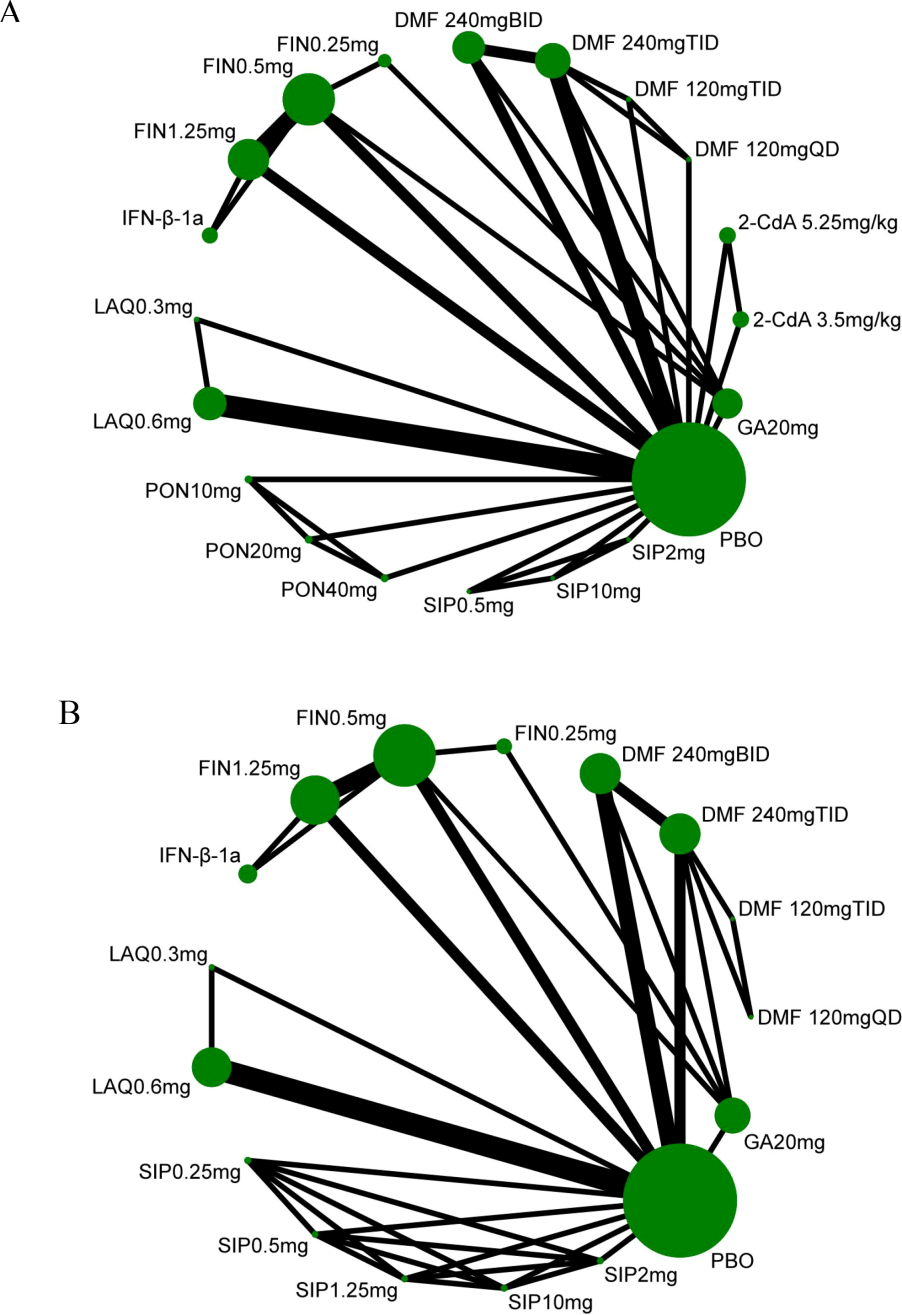
Evidence network diagram of ARR **(A)**; DAE **(B)**. The size of nodes is proportional to the number of participants (sample size), and the width of lines is proportional to the number of interventions between two points (number of study groups/arms).

**Figure 3** presents the evidence network diagrams for the efficacy outcome ARR and safety outcome DAE. The results demonstrate that the ARR analysis incorporated 14 RCTs involving 13,315 patients. With the exception of FIN 0.25 mg which lacked direct comparison with placebo, all other interventions had at least one placebo-controlled trial. Additionally, FIN 0.5 mg and FIN 1.25 mg were directly compared with the active comparator IFN-β-1a, while FIN 0.25 mg, FIN 0.5 mg, DMF 240 mg BID, and DMF 240 mg TID were directly compared with the active comparator GA 20 mg.

For the DAE outcome, 12 RCTs involving 10,710 patients were included. Except for DMF 120 mg QD, DMF 120 mg TID, and FIN 0.25 mg which had no direct placebo comparisons, all other interventions included at least one placebo-controlled trial. Furthermore, FIN 0.5 mg and FIN 1.25 mg were directly compared with IFN-β-1a, while FIN 0.25 mg, FIN 0.5 mg, DMF 240 mg BID, and DMF 240 mg TID were directly compared with GA 20 mg.

#### 3.3.3 Network meta-analysis

Regarding the primary efficacy endpoint ARR, standard treatment regimens including Siponimod 2 mg [MD = -0.38, 95% CI (-0.76, 0.00)], Fingolimod 0.5 mg [MD = -0.21, 95% CI (-0.25, -0.17)], Cladribine 3.5 mg/kg [MD = -0.19, 95% CI (-0.24, -0.14)], Dimethyl Fumarate 240 mg BID [MD = -0.19, 95% CI (-0.24, -0.13)], Laquinimod 0.6 mg [MD = -0.09, 95% CI (-0.13, -0.04)], and GA20mg [MD = -0.10, 95% CI (-0.16, -0.05)] demonstrated statistically significant superiority over PBO (**Supplementary Table 15**).

For the primary safety endpoint DAE, LAQ0.6mg [RR = 0.64, 95% CI (0.44,0.94)] showed statistically significant differences compared with PBO (**Supplementary Table 16**).

#### 3.3.4 Ranking of the results of reticulated meta-analysis

**Figure 4:**
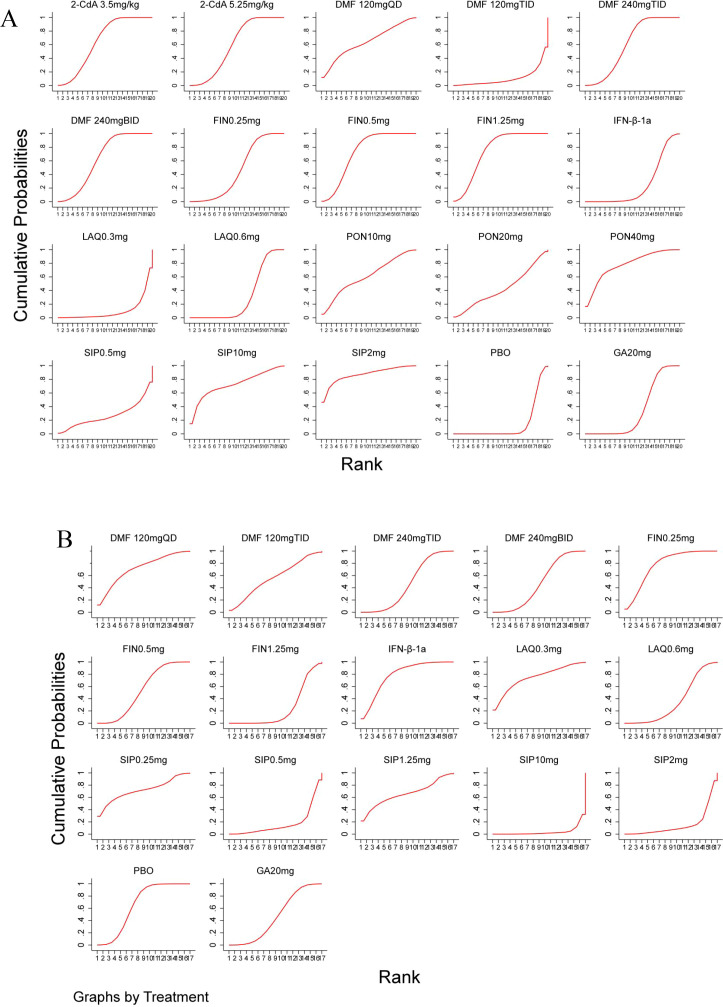
The SUCRA ranking diagram of ARR **(A)** and DAE **(B)**.

For ARR, the standard treatment regimens were ranked from best to worst in terms of therapeutic efficacy as follows: SIP 2 mg (SUCRA = 86.9%) > FIN 0.5 mg (SUCRA = 72.6%) > 2-CdA 3.5 mg/kg (SUCRA = 65.2%) > DMF 240 mg BID (SUCRA = 63.1%) > PON 10 mg (SUCRA = 61.1%) > PON 20 mg (SUCRA = 42.9%) > GA20mg (SUCRA = 34.4%) > LAQ 0.6 mg (SUCRA = 30.5%) > SIP 0.5 mg (SUCRA = 26.9%) > IFN-β-1a (SUCRA = 25.5%) > PBO (SUCRA = 14.2%) (**Figure 4A**).

For DAE, the standard treatment regimens were ranked from best to worst in terms of safety as follows: IFN-β-1a 30 µg (SUCRA = 82.7%) > SIP 0.25 mg (SUCRA = 70.1%) > FIN 0.5 mg (SUCRA = 68.7%) > SIP 1.25 mg (SUCRA = 65.3%) > PBO (SUCRA = 59.9%) > GA20mg (SUCRA = 50.9%) > DMF 240 mg BID (SUCRA = 43.5%) > LAQ 0.6 mg (SUCRA = 30.5%) > SIP 0.5 mg (SUCRA = 15.5%) > SIP 2 mg (SUCRA = 13.9%) (**Figure 4B**).

Additionally, we created a two-dimensional ranking plot to evaluate the SUCRA values for the efficacy outcome ARR and the safety outcome DAE, which provides a more intuitive and clear visualization of the efficacy and safety outcomes of different interventions. Since 2-CdA 3.5mg/kg, 2-CdA 5.25mg/kg, PON10mg, PON20mg, and PON40mg lacked suitable inclusion data for DAE, and SIP0.25mg and SIP1.25mg lacked suitable inclusion data for ARR, the two-dimensional plot included a total of 15 interventions (**Supplementary Figure 2**).

#### 3.3.5 Inconsistency test

**Figure 5:**
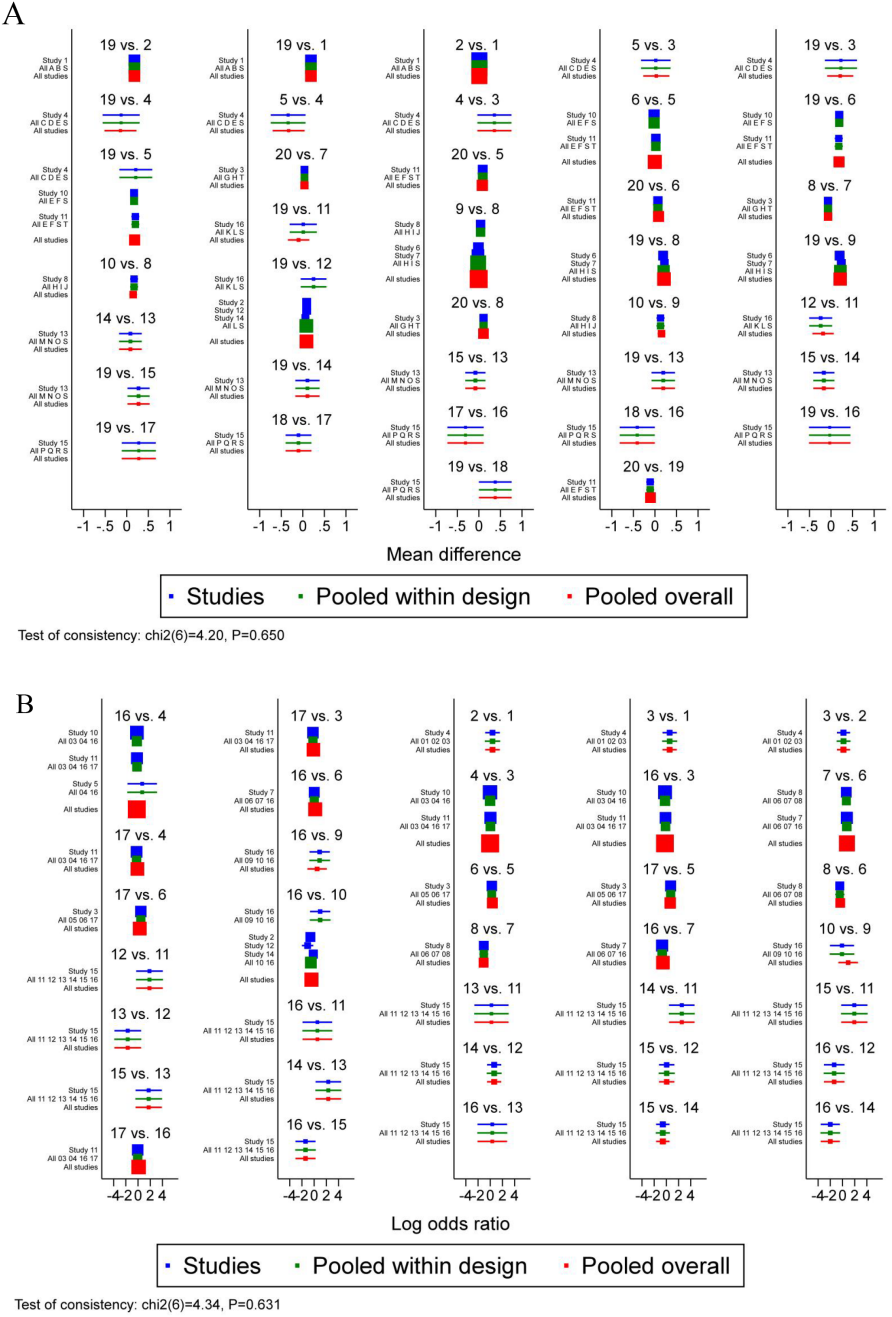
Overall inconsistency test of ARR **(A)** and DAE **(B)**.

Through the global inconsistency test, no significant differences were observed between the consistency and inconsistency models for ARR (P = 0.650) (**Figure 5A**) and DAE (P = 0.631) (**Figure 5B**). The loop inconsistency test demonstrated consistency across all closed loops, with ARR’s 95% CI lower bound including 0 (P > 0.05) and tau² value approximating 0 (**Supplementary Table 1**), and DAE’s 95% CI lower bound including 0 (P > 0.05) with tau² value also approximating 0 (**Supplementary Table 2**). The node-splitting method results indicated no statistically significant differences between direct and indirect comparisons for each split node in ARR (**Supplementary Table 3**) and DAE (**Supplementary Table 4**) (P > 0.05), suggesting consistency in comparisons between any two interventions and the feasibility of result pooling.

#### 3.3.6 Publication bias

**Figure 6:**
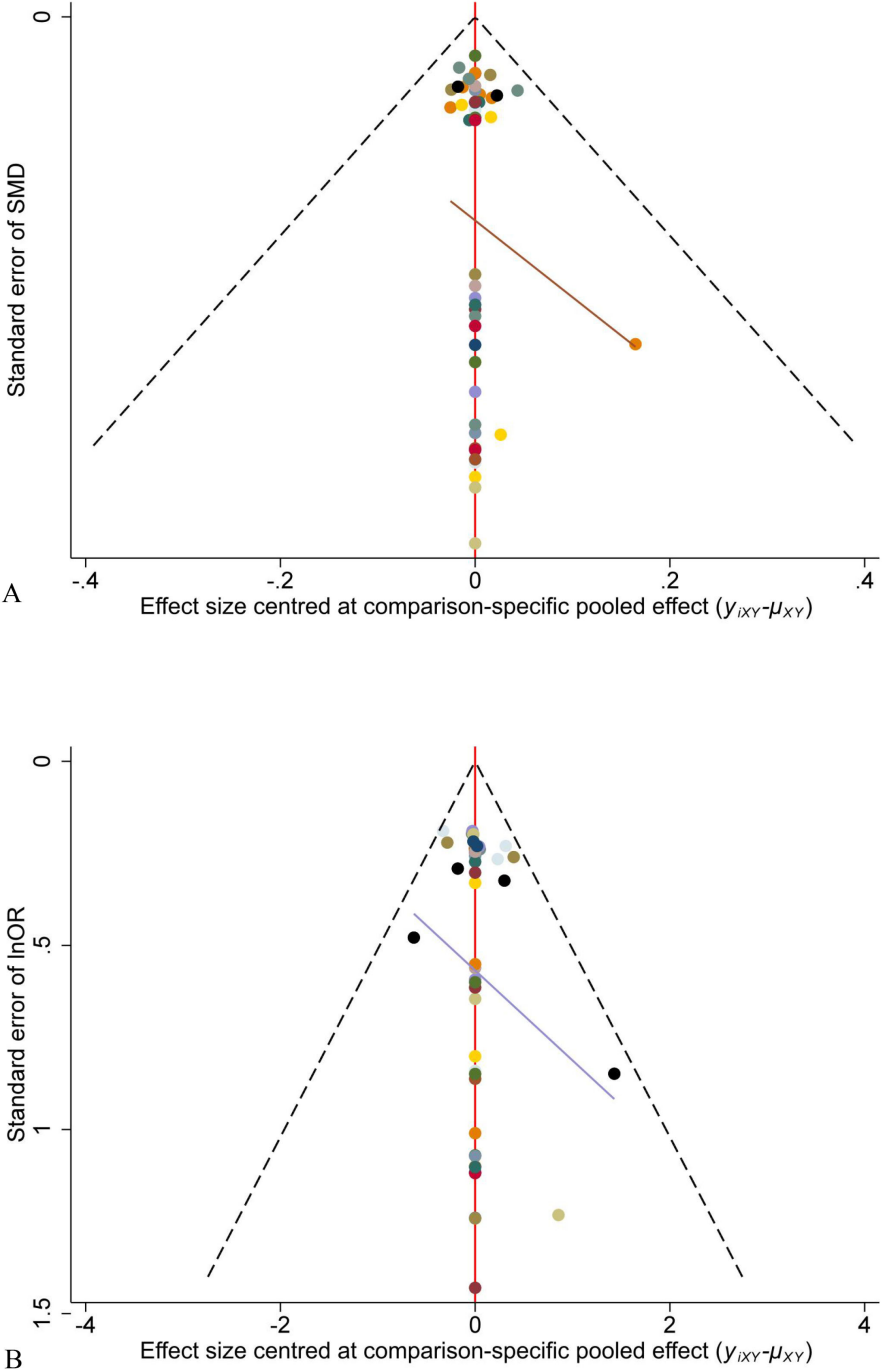
Comparison-adjusted funnel plots of ARR **(A)** and DAE **(B)**.

The distribution of included studies on both sides of the centerline was essentially symmetrical, indicating minimal publication bias. Some studies were located below the inverted funnel plot with larger standard errors, suggesting the potential presence of a small sample size effect (**Figure 6**).

### 3.4 Secondary outcome measures

#### 3.4.1 Pairwise meta-analysis

T1 and T2 were analyzed using a fixed-effects model for meta-analysis. Since there were no direct comparisons between studies, heterogeneity testing was not performed.

Adverse events (AE) were analyzed using a random-effects model for meta-analysis, reflecting the impact of 19 interventions on AEs. Low heterogeneity was observed between studies (I² ≤ 46%). The traditional meta-analysis results showed that the risk of AEs was significantly higher with DMF 240mg BID compared to placebo (PBO) [OR = 1.56, 95% CI (1.08, 2.27)], and significantly higher with LAQ 0.6mg compared to PBO [OR = 1.26, 95% CI (1.00, 1.59)] (**Supplementary Figure 3**).Serious adverse events (SAE) were analyzed using a random-effects model for meta-analysis, reflecting the impact of 17 interventions on SAEs. Low to moderate heterogeneity was observed between studies (I² ≤ 62%) (**Supplementary Figure 4**).

#### 3.4.2 Evidence network diagram

For the secondary outcome measure T1, a total of 2 RCTs were included (2 four-arm studies and 1 three-arm study), involving 529 patients (**Supplementary Figure 5**). For T2, 2 four-arm RCTs were included with 612 patients. Each intervention was directly compared with placebo (**Supplementary Figure 6**).

A total of 15 RCTs were included for AEs (2 two-arm, 10 three-arm, and 4 multi-arm studies), involving 13,639 patients (**Supplementary Figure 7**). For SAEs, 13 RCTs were included (2 two-arm, 10 three-arm, and 3 multi-arm studies), involving 13,383 patients (**Supplementary Figure 8**). In these two outcome measures, FIN 0.25 mg and FIN 0.5 mg were directly compared head-to-head, without a direct comparison between FIN 0.25 mg and PBO. Additionally, FIN 0.5 mg and FIN 1.25 mg were directly compared with the active comparator IFN-β-1a, while FIN 0.25 mg, FIN 0.5 mg, DMF 240 mg BID, DMF 240 mg TID, and GA 20 mg were directly compared with the active control drug.

#### 3.4.3 Network meta-analysis

The secondary efficacy outcome measure T1 demonstrated that the non-standard treatment regimen DMF 240mg TID [MD = -0.90, 95% CI (-1.75, -0.05)] was significantly superior to PBO (**Supplementary Table 5**); no statistically significant comparisons were found in the T2 analysis results (**Supplementary Table 6**).

The secondary safety outcome measure AE indicated that PBO showed differences compared to the standard treatment regimens LAQ 0.6mg [MD = 0.78, 95% CI (0.66, 0.91)], 2-CdA 3.5mg/kg [MD = 0.66, 95% CI (0.48, 0.91)], DMF 240mg BID [MD = 0.6, 95% CI (0.42, 0.86)], and SIP 2mg [MD = 0.09, 95% CI (0.01, 0.68)], suggesting potential safety concerns for the latter (**Supplementary Table 7**).

The SAE analysis results revealed a significant difference between PBO and the standard treatment regimen SIP 0.5mg [RR = 0.03, 95% CI (0.00, 0.61)], indicating potential safety risks for the latter (**Supplementary Table 8**).

#### 3.4.4 Ranking of the results of reticulated meta-analysis

Secondary efficacy outcome measures for standard treatment regimens were ranked as follows for T1: LAQ 0.6mg (SUCRA = 67.9%) > PBO (SUCRA = 28.8%) (**Supplementary Figure 9**); for T2: PON 20mg (SUCRA = 65.2%) > PON 10mg (SUCRA = 41.2%) > PBO (SUCRA = 20.1%) (**Supplementary Figure 10**).

For secondary safety outcome measures regarding standard treatment regimens, the SUCRA rankings for AEs were: SIP 1.25mg (SUCRA = 92.3%) > GA 20mg (SUCRA = 90.6%) > SIP 0.25mg (SUCRA = 83.5%) > PBO (SUCRA = 73%) > FIN 0.5mg (SUCRA = 66.3%) > PON 10mg (SUCRA = 59.5%) > PON 20mg (SUCRA = 58.1%) > LAQ 0.6mg (SUCRA = 51.4%) > SIP 0.5mg (SUCRA = 42.5%) > 2-CDA 3.5mg/kg (SUCRA = 38.2%) > DMF 240mg BID (SUCRA = 31.1%) > IFN-β-1a (SUCRA = 28.9%) > SIP 2mg (SUCRA = 4%) (**Supplementary Figure 11**). The rankings for SAEs were: IFN-β-1a (SUCRA = 87.8%) > GA 20mg (SUCRA = 79%) > DMF 240mg BID (SUCRA = 76%) > FIN 0.5mg (SUCRA = 68.4%) > PBO (SUCRA = 57.7%) > SIP 0.25mg (SUCRA = 56.9%) > LAQ 0.6mg (SUCRA = 47.1%) > PON 20mg (SUCRA = 41.2%) > 2-CdA 3.5mg/kg (SUCRA = 41%) > PON 10mg (SUCRA = 38.6%) > SIP 1.25mg (SUCRA = 21.8%) > SIP 2mg (SUCRA = 13.3%) > SIP 0.5mg (SUCRA = 2.2%) (**Supplementary Figure 12**).

#### 3.4.5 Inconsistency test

Secondary efficacy outcome T1 (P = 0.518) could not undergo loop inconsistency testing due to the limited number of included studies, but other inconsistency tests remained necessary to conduct, with results still holding certain significance. The global inconsistency test revealed no significant differences between consistency and inconsistency models (**Supplementary Figure 13**). The node-splitting method results indicated no statistically significant differences between direct and indirect comparison outcomes at each split node (P > 0.05), suggesting good consistency in comparisons between any two interventions, with favorable results suitable for merging (**Supplementary Table 5**).

The global inconsistency test for T2 (P = 0.352) showed no significant difference between consistency and inconsistency models (**Supplementary Figure 14**). Due to the limited number of included studies, the loop inconsistency test could not be performed. The node-splitting method results indicated no statistically significant differences between direct and indirect comparisons at each split node (P > 0.05), suggesting good consistency in comparisons between any two interventions and supporting the validity of result synthesis (**Supplementary Table 6**).

Secondary safety outcome analysis of adverse events (AE, P = 0.199) demonstrated no significant differences between consistency and inconsistency models in the global inconsistency test (**Supplementary Figure 15**). Similarly, the loop inconsistency test indicated all closed loops were consistent, with 95% CIs including 0 (P>0.05) and tau² values approximating 0 (**Supplementary Table 7**). The node-splitting method revealed no statistically significant differences between direct and indirect comparisons at any split nodes (P>0.05), suggesting consistency in all pairwise treatment comparisons and supporting the validity of pooled results (**Supplementary Table 8**).

The global inconsistency test for SAE (P = 0.786) showed no significant difference between consistency and inconsistency models (**Supplementary Figure 16**); similarly, the loop inconsistency test indicated that all closed loops were consistent, with 95% CI including zero, P > 0.05, and tau² value approximating 0 (**Supplementary Table 9**); the node-splitting method demonstrated no statistically significant differences between direct and indirect comparisons at each split node (P > 0.05), suggesting that comparisons between any two interventions were consistent and the results could be appropriately pooled (**Supplementary Table 10**).

#### 3.4.6 Publication bias

Due to the limited number of included studies (fewer than 10) in T1 and T2, which is insufficient to effectively demonstrate potential bias, funnel plots were not constructed.

The funnel plot for AE adjustment demonstrated a generally symmetrical distribution of included studies on both sides of the centerline, suggesting a low probability of publication bias. A small number of studies located below the funnel plot exhibited larger standard errors, indicating the possible presence of a small sample size effect (**Supplementary Figure 17**).

The funnel plot of SAE correction demonstrates that the studies are predominantly distributed along the symmetry line, indicating a low likelihood of bias. However, some studies are located below the funnel plot with substantial standard errors, suggesting the potential presence of a small sample size effect (**Supplementary Figure 18**).

## 4 Discussion

This network meta-analysis conducted a comparative estimation and ranking of the efficacy and safety profiles of various oral medications for relapsing-remitting multiple sclerosis (RRMS) by integrating both direct and indirect evidence, thereby providing evidence-based references for the pharmacological treatment of this disease. Initially, the study planned to investigate 10 oral disease-modifying therapies (DMTs), but systematic literature search and screening revealed only 6 drugs with relevant research publications on RRMS treatment, while no applicable research data were found for the remaining 4 drugs.The results demonstrated that SIP 2 mg (MD = -0.38, 95% CI: -0.76, 0.00) exhibited superior efficacy in reducing annualized relapse rate (ARR), with a SUCRA value reaching 86.9%, outperforming most other medications. This finding aligns with previous studies (32, 33), suggesting that SIP may effectively suppress disease activity through its unique pharmacological mechanism (preventing synaptic neurodegeneration and promoting remyelination) (34).Regarding comparisons between different dosages and administration frequencies of DMF, while increased dosage and frequency showed trends toward reduced relapse rates, no statistically significant differences were observed. Research indicates this may relate to DMF’s neurofunctional improvement via Nrf2 pathway activation, along with its neuroprotective effects in suppressing reactive oxygen species generation and apoptosis (35–37).Notably, this study incorporated exploratory data from non-standard regimens such as DMF 120mg QD and SIP 10mg. These data primarily serve to comprehensively depict dose-response curves within statistical models and provide exploratory references for subsequent dose-related research, rather than representing clinically approved standard regimens. Analytical results derived from such data require cautious interpretation and should not be used as decision-making basis for routine clinical practice.

This study systematically reviews the core safety monitoring requirements for various oral disease-modifying therapies in RRMS as documented in included studies and drug prescribing information. For all investigational drugs, complete blood counts (including lymphocyte counts) should be routinely performed throughout the treatment course, accompanied by continuous screening for infection symptoms, constituting fundamental safety measures for long-term clinical medication. Additionally, drug-specific safety monitoring points require particular attention: ECG monitoring is mandatory prior to initiating siponimod and ponesimod therapy, with first-dose monitoring required for patients with cardiac comorbidities, while siponimod is contraindicated in patients carrying the CYP2C933 genotype; cladribine necessitates additional hepatic and renal function monitoring, supplemented with immune function testing and tumor screening for long-term users; laquinimod requires liver function parameters as primary monitoring indicators while warranting vigilance against infection risks associated with induced hypogammaglobulinemia; although fingolimod demonstrates relatively favorable safety profiles, pre-treatment cardiac evaluation and complete blood counts remain essential, with first-dose monitoring required during initial administration.

This study possesses methodological advantages that address the limitations of traditional meta-analyses which could only perform direct comparisons. Through rigorous literature screening and quality assessment, the reliability of included studies was ensured. The search strategy aimed to cover all relevant oral disease-modifying treatment drugs to enhance the analysis’s reliability. Although a few interventions met the screening criteria, no relevant clinical trials were retrieved, resulting in inconsistency between the search strategy and the final number of included interventions. Furthermore, the robustness of the results was verified through consistency testing and publication bias analysis.

This study has certain limitations. Firstly, some research samples were relatively small, which may lead to less precise effect estimates and increased uncertainty in the results. For instance, the SIP 0.5 mg group had only 43 cases, potentially providing insufficient statistical power when analyzing certain indicators or rare outcomes. Additionally, we examined heterogeneity among direct comparisons through meta-analysis. For a small number of outcome indicators with significant heterogeneity, although a random-effects model was employed, certain inter-study heterogeneity remained (with I²≤50% in the vast majority of studies). Studies with I²>50% that could not be reduced to I²≤50% through random-effects analysis were excluded. Our meta-analysis explored some sources of heterogeneity, revealing that it might stem from racial differences among patients across studies, variations in the assessment criteria for severe adverse reactions, and differences in specific details of drug administration. While unidentified confounding factors may still exist, they do not affect the stability of the results. For example, in the traditional meta-comparison of DAE, the substantial heterogeneity in FIN 0.5mg VS PBO might be due to differences in the implementation of adverse event definitions between two trials. Kappos L, 2010 (23)adopted stricter discontinuation criteria for specific events, leading to the conversion of the 1.25 mg group to the 0.5 mg group midway through the trial (November 2009) due to safety concerns, while still analyzing it as the original group during statistical analysis, which might have influenced the final results. In contrast, Calabresi PA, 2013 (24)did not mandate discontinuation due to laboratory abnormalities. Finally, although the two studies had different discontinuation rates, both confirmed that fingolimod 0.5 mg significantly reduced relapse rates and brain atrophy. The safety differences primarily arose from study design rather than inherent changes in the drug itself, hence these studies were not excluded. The fingolimod prescribing information specifies a dose of 0.5 mg and notes that higher doses provide no additional benefit and are associated with increased adverse reaction rates, which aligns with the findings of this study.

The data integrity requires further improvement, as significant variations and missing data were observed in some secondary outcome measures such as sustained disability progression during statistical analysis, which affected the assessment of drug safety and efficacy. Additionally, most included studies had relatively short follow-up periods, potentially compromising the accuracy of long-term efficacy and safety evaluations for this chronic disease, RRMS. Finally, despite employing multiple search strategies, publication bias could not be completely ruled out, as studies with negative results might have been insufficiently included, introducing a certain degree of bias to the findings.

To address the limitations of this study, future research could be conducted from multiple perspectives. To enhance study accuracy, large-scale, multicenter randomized controlled trials should be implemented, with rigorous standardization of study design and execution processes to ensure broad representativeness of samples and standardization of interventions. For instance, during patient recruitment, explicit inclusion of patients from diverse ethnic backgrounds and different disease stages should be ensured, along with uniform drug dosage, treatment duration, and measurement methods for observation indicators. Meanwhile, follow-up periods should be appropriately extended to more accurately assess the long-term efficacy and safety of medications. Furthermore, future studies could expand their scope to explore the efficacy and safety of different oral medications in special populations such as children, elderly individuals, and patients with comorbid conditions. Research could also investigate combination therapies involving multiple drugs to provide more treatment options for RRMS. Methodologically, more advanced statistical models, such as Bayesian network meta-analysis, could be employed to better address inconsistencies between direct and indirect evidence and improve the reliability of analytical results.

## 5 Conclusions

This study conducted a network meta-analysis to comparatively estimate the efficacy and safety of multiple oral medications for RRMS, demonstrating that SIP 2 mg significantly reduced ARR. FIN 0.25 mg exhibited relative safety advantages in terms of DAE, though this dosage represents an exploratory regimen and should not serve as a basis for routine clinical use. The research strictly differentiated between clinically approved standard doses and exploratory non-standard doses, providing evidence-based references for developing individualized RRMS treatment plans, policymaking by healthcare authorities, and drug development. While this study has certain limitations, future high-quality research is anticipated to further refine the evidence-based system for RRMS therapeutics, offering patients superior treatment options.

## Data Availability

The original contributions presented in the study are included in the article/**Supplementary Material**. Further inquiries can be directed to the corresponding author.

## Glossary

AE: Adverse events
ARR: annualized relapse rate
2-CdA 3.5mg/kg: Cladribine 3.5 mg/kg
2-CdA 5.25mg/kg: Cladribine 5.25 mg/kg
CNS: central nervous system
DAE: Adverse events leading to discontinuation
DMDs: Disease-Modifying Drugs
DMF 120mgQD: Dimethyl Fumarate 120 mg once daily
DMF 120mgTID: Dimethyl Fumarate 120 mg three times daily
DMF 240mgTID: Dimethyl Fumarate 240 mg three times daily
DMF 240mgBID: Dimethyl Fumarate 240 mg twice Daily
DMTs: Disease-Modifying Therapies
DRF: Diroximel fumarate
FIN0.25mg: Fingolimod 0.25 mg
FIN0.5mg: Fingolimod 0.5 mg
FIN1.25mg: Fingolimod 1.25 mg
IF: Inconsistency factors
IFN-β-1a: Interferon β-1a 30 µg
LAQ0.3mg: Laquinimod 0.3 mg
LAQ0.6mg: Laquinimod 0.6 mg
MD: Mean difference
MRI: magnetic resonance imaging
MS: multiple sclerosis
OR: Odds ratio
Oza: Ozanimod
PBO: Placebo
PON10mg: Ponesimod 10 mg
PON20mg: Ponesimod 20 mg
PON40mg: Ponesimod 40 mg
RCT: randomized controlled trial
RRMS: Relapsing-Remitting Multiple Sclerosis
SAE: serious adverse event
SE: Standard error
SIP0.5mg: Siponimod 0.5 mg
SIP10mg: Siponimod 10mg
SIP2mg: Siponimod 2 mg
SUCRA: Surface under the cumulative ranking
TER: Teriflunomide;

## References

[1] Mey GM Mahajan KR DeSilva TM . Neurodegeneration in multiple sclerosis. WIREs Mech disease. (2023) 15:e1583. doi: 10.1002/wsbm.1583, PMID: 35948371 PMC9839517

[2] Ontaneda D Thompson AJ Fox RJ Cohen JA . Progressive multiple sclerosis: prospects for disease therapy, repair, and restoration of function. Lancet (London England). (2017) 389:1357–66. doi: 10.1016/S0140-6736(16)31320-4, PMID: 27889191

[3] Tramacere I Del Giovane C Salanti G D’Amico R Filippini G . Immunomodulators and immunosuppressants for relapsing-remitting multiple sclerosis: a network meta-analysis. Cochrane Database systematic Rev. (2015) 2015:Cd011381. doi: 10.1002/14651858.CD011381.pub2, PMID: 26384035 PMC9235409

[4] Psenicka MW Smith BC Tinkey RA Williams JL . Connecting neuroinflammation and neurodegeneration in multiple sclerosis: are oligodendrocyte precursor cells a nexus of disease? Front Cell Neurosci. (2021) 15:654284. doi: 10.3389/fncel.2021.654284, PMID: 34234647 PMC8255483

[5] Gold R Kappos L Arnold DL Bar-Or A Giovannoni G Selmaj K . Placebo-controlled phase 3 study of oral BG-12 for relapsing multiple sclerosis. New Engl J Med. (2012) 367:1098–107. doi: 10.1056/NEJMoa1114287, PMID: 22992073

[6] Harrison J Hill J Palmer K . Disease modifying therapies for multiple sclerosis: benefit and acceptability. Br J Neurosci Nurs. (2022) 18:S16–s9. doi: 10.12968/bjnn.2022.18.Sup3.S16, PMID: 38213413 PMC7615514

[7] Bar-Or A Pachner A Menguy-Vacheron F Kaplan J Wiendl H . Teriflunomide and its mechanism of action in multiple sclerosis. Drugs. (2014) 74:659–74. doi: 10.1007/s40265-014-0212-x, PMID: 24740824 PMC4003395

[8] Subei AM Cohen JA . Sphingosine 1-phosphate receptor modulators in multiple sclerosis. CNS Drugs. (2015) 29:565–75. doi: 10.1007/s40263-015-0261-z, PMID: 26239599 PMC4554772

[9] Chun J Giovannoni G Hunter SF . Sphingosine 1-phosphate receptor modulator therapy for multiple sclerosis: differential downstream receptor signalling and clinical profile effects. Drugs. (2021) 81:207–31. doi: 10.1007/s40265-020-01431-8, PMID: 33289881 PMC7932974

[10] Yadav SK Soin D Ito K Dhib-Jalbut S . Insight into the mechanism of action of dimethyl fumarate in multiple sclerosis. J Mol Med (Berlin Germany). (2019) 97:463–72. doi: 10.1007/s00109-019-01761-5, PMID: 30820593

[11] Comi G Hartung HP Kurukulasuriya NC Greenberg SJ Scaramozza M . Cladribine tablets for the treatment of relapsing-remitting multiple sclerosis. Expert Opin pharmacotherapy. (2013) 14:123–36. doi: 10.1517/14656566.2013.754012, PMID: 23256518

[12] Engel S Jolivel V Kraus SH Zayoud M Rosenfeld K Tumani H . Laquinimod dampens IL-1β signaling and Th17-polarizing capacity of monocytes in patients with MS. Neurology(R) neuroimmunology Neuroinflamm. (2021) 8. doi: 10.1212/NXI.0000000000000908, PMID: 33203651 PMC7676421

[13] Reilmann R Anderson KE Feigin A Tabrizi SJ Leavitt BR Stout JC . Safety and efficacy of laquinimod for Huntington’s disease (LEGATO-HD): a multicentre, randomised, double-blind, placebo-controlled, phase 2 study. Lancet Neurology. (2024) 23:243–55. doi: 10.1016/S1474-4422(23)00454-4, PMID: 38280392

[14] Varrin-Doyer M Zamvil SS Schulze-Topphoff U . Laquinimod, an up-and-coming immunomodulatory agent for treatment of multiple sclerosis. Exp Neurol. (2014) 262 Pt A:66–71. doi: 10.1016/j.expneurol.2014.04.002, PMID: 24731945 PMC4195809

[15] Bross M Hackett M Bernitsas E . Approved and emerging disease modifying therapies on neurodegeneration in multiple sclerosis. Int J Mol Sci. (2020) 21. doi: 10.3390/ijms21124312, PMID: 32560364 PMC7348940

[16] Lee CY Chan KH . Personalized use of disease-modifying therapies in multiple sclerosis. Pharmaceutics. (2024) 16. doi: 10.3390/pharmaceutics16010120, PMID: 38258130 PMC10820407

[17] Filippi M Danesi R Derfuss T Duddy M Gallo P Gold R . Early and unrestricted access to high-efficacy disease-modifying therapies: a consensus to optimize benefits for people living with multiple sclerosis. J neurology. (2022) 269:1670–7. doi: 10.1007/s00415-021-10836-8, PMID: 34626224 PMC8501364

[18] Giovannoni G Comi G Cook S Rammohan K Rieckmann P Soelberg Sørensen P . A placebo-controlled trial of oral cladribine for relapsing multiple sclerosis. New Engl J Med. (2010) 362:416–26. doi: 10.1056/NEJMoa0902533, PMID: 20089960

[19] Kappos L Gold R Miller DH Macmanus DG Havrdova E Limmroth V . Efficacy and safety of oral fumarate in patients with relapsing-remitting multiple sclerosis: a multicentre, randomised, double-blind, placebo-controlled phase IIb study. Lancet (London England). (2008) 372:1463–72. doi: 10.1016/S0140-6736(08)61619-0, PMID: 18970976

[20] Fox RJ Miller DH Phillips JT Hutchinson M Havrdova E Kita M . Placebo-controlled phase 3 study of oral BG-12 or glatiramer in multiple sclerosis. New Engl J Med. (2012) 367:1087–97. doi: 10.1056/NEJMoa1206328, PMID: 22992072

[21] Saida T Yamamura T Kondo T Yun J Yang M Li J . A randomized placebo-controlled trial of delayed-release dimethyl fumarate in patients with relapsing-remitting multiple sclerosis from East Asia and other countries. BMC neurology. (2019) 19:5. doi: 10.1186/s12883-018-1220-3, PMID: 30616596 PMC6322309

[22] Cohen JA Barkhof F Comi G Hartung HP Khatri BO Montalban X . Oral fingolimod or intramuscular interferon for relapsing multiple sclerosis. New Engl J Med. (2010) 362:402–15. doi: 10.1056/NEJMoa0907839, PMID: 20089954

[23] Kappos L Radue EW O’Connor P Polman C Hohlfeld R Calabresi P . A placebo-controlled trial of oral fingolimod in relapsing multiple sclerosis. New Engl J Med. (2010) 362:387–401. doi: 10.1056/NEJMoa0909494, PMID: 20089952

[24] Calabresi PA Radue EW Goodin D Jeffery D Rammohan KW Reder AT . Safety and efficacy of fingolimod in patients with relapsing-remitting multiple sclerosis (FREEDOMS II): a double-blind, randomised, placebo-controlled, phase 3 trial. Lancet Neurology. (2014) 13:545–56. doi: 10.1016/S1474-4422(14)70049-3, PMID: 24685276

[25] Cree BAC Goldman MD Corboy JR Singer BA Fox EJ Arnold DL . Efficacy and safety of 2 fingolimod doses vs glatiramer acetate for the treatment of patients with relapsing-remitting multiple sclerosis: A randomized clinical trial. JAMA neurology. (2020) 78:1–13. doi: 10.1001/jamaneurol.2020.2950, PMID: 32852530 PMC7445630

[26] Comi G Pulizzi A Rovaris M Abramsky O Arbizu T Boiko A . Effect of laquinimod on MRI-monitored disease activity in patients with relapsing-remitting multiple sclerosis: a multicentre, randomised, double-blind, placebo-controlled phase IIb study. Lancet (London England). (2008) 371:2085–92. doi: 10.1016/S0140-6736(08)60918-6, PMID: 18572078

[27] Comi G Jeffery D Kappos L Montalban X Boyko A Rocca MA . Placebo-controlled trial of oral laquinimod for multiple sclerosis. New Engl J Med. (2012) 366:1000–9. doi: 10.1056/NEJMoa1104318, PMID: 22417253

[28] Vollmer TL Sorensen PS Selmaj K Zipp F Havrdova E Cohen JA . A randomized placebo-controlled phase III trial of oral laquinimod for multiple sclerosis. J neurology. (2014) 261:773–83. doi: 10.1007/s00415-014-7264-4, PMID: 24535134

[29] Comi G Dadon Y Sasson N Steinerman JR Knappertz V Vollmer TL . CONCERTO: A randomized, placebo-controlled trial of oral laquinimod in relapsing-remitting multiple sclerosis. Multiple sclerosis (Houndmills Basingstoke England). (2022) 28:608–19. doi: 10.1177/13524585211032803, PMID: 34378456

[30] Olsson T Boster A Fernández Ó Freedman MS Pozzilli C Bach D . Oral ponesimod in relapsing-remitting multiple sclerosis: a randomised phase II trial. J neurology neurosurgery Psychiatry. (2014) 85:1198–208. doi: 10.1136/jnnp-2013-307282, PMID: 24659797 PMC4215282

[31] Selmaj K Li DK Hartung HP Hemmer B Kappos L Freedman MS . Siponimod for patients with relapsing-remitting multiple sclerosis (BOLD): an adaptive, dose-ranging, randomised, phase 2 study. Lancet Neurology. (2013) 12:756–67. doi: 10.1016/S1474-4422(13)70102-9, PMID: 23764350

[32] Faissner S Gold R . Efficacy and safety of multiple sclerosis drugs approved since 2018 and future developments. CNS Drugs. (2022) 36:803–17. doi: 10.1007/s40263-022-00939-9, PMID: 35869335 PMC9307218

[33] Cree BA Arnold DL Fox RJ Gold R Vermersch P Benedict RH . Long-term efficacy and safety of siponimod in patients with secondary progressive multiple sclerosis: Analysis of EXPAND core and extension data up to >5 years. Multiple sclerosis (Houndmills Basingstoke England). (2022) 28:1591–605. doi: 10.1177/13524585221083194, PMID: 35380078 PMC9315196

[34] Vališ M Achiron A Hartung HP Mareš J Tichá V Štourač P . The benefits and risks of switching from fingolimod to siponimod for the treatment of relapsing-remitting and secondary progressive multiple sclerosis. Drugs R&D. (2023) 23:331–8. doi: 10.1007/s40268-023-00434-6, PMID: 37640862 PMC10676342

[35] Linker RA Lee DH Ryan S van Dam AM Conrad R Bista P . Fumaric acid esters exert neuroprotective effects in neuroinflammation via activation of the Nrf2 antioxidant pathway. Brain: J Neurol. (2011) 134:678–92. doi: 10.1093/brain/awq386, PMID: 21354971

[36] Zhao X Sun G Zhang J Ting SM Gonzales N Aronowski J . Dimethyl fumarate protects brain from damage produced by intracerebral hemorrhage by mechanism involving Nrf2. Stroke. (2015) 46:1923–8. doi: 10.1161/STROKEAHA.115.009398, PMID: 25977275 PMC4480061

[37] Wang Q Chuikov S Taitano S Wu Q Rastogi A Tuck SJ . Dimethyl fumarate protects neural stem/progenitor cells and neurons from oxidative damage through Nrf2-ERK1/2 MAPK pathway. Int J Mol Sci. (2015) 16:13885–907. doi: 10.3390/ijms160613885, PMID: 26090715 PMC4490529

