# Supplementary material for: Efficacy and safety of disease-modifying oral drugs in treatment of relapsing-remitting multiple sclerosis: systematic review and network meta-analysis
Source: Front Immunol. 2026 Mar 16;17:1733948. doi: 10.3389/fimmu.2026.1733948 (PMC13033576; doi:10.3389/fimmu.2026.1733948)

**Supplementary Figure1(A):Pairwise Meta‑analysis for ARR**


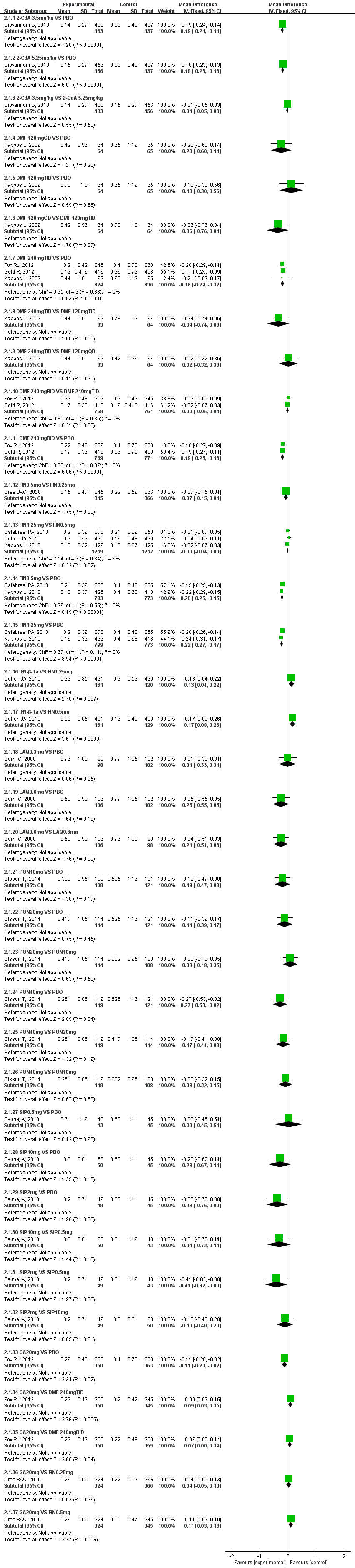


**Supplementary Figure1(B):Pairwise Meta‑analysis for DAE**


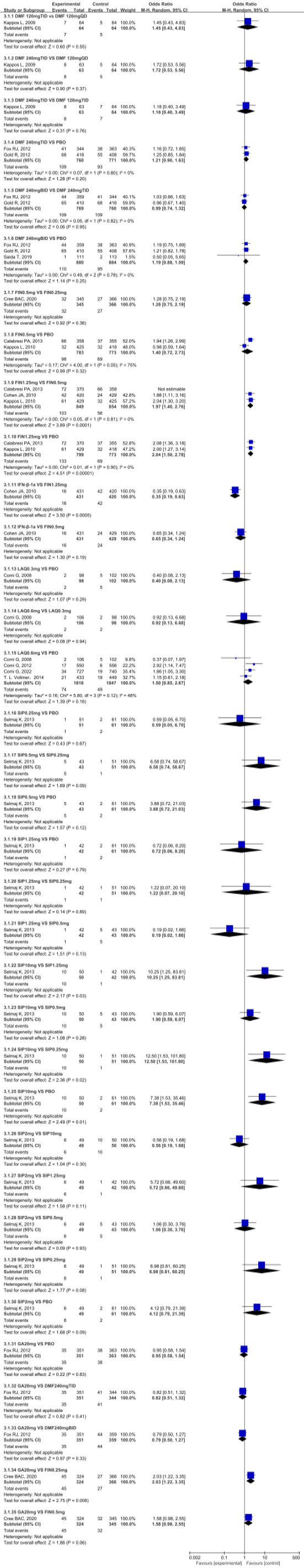


**Supplementary Figure 2:two-dimensional ranking plot**


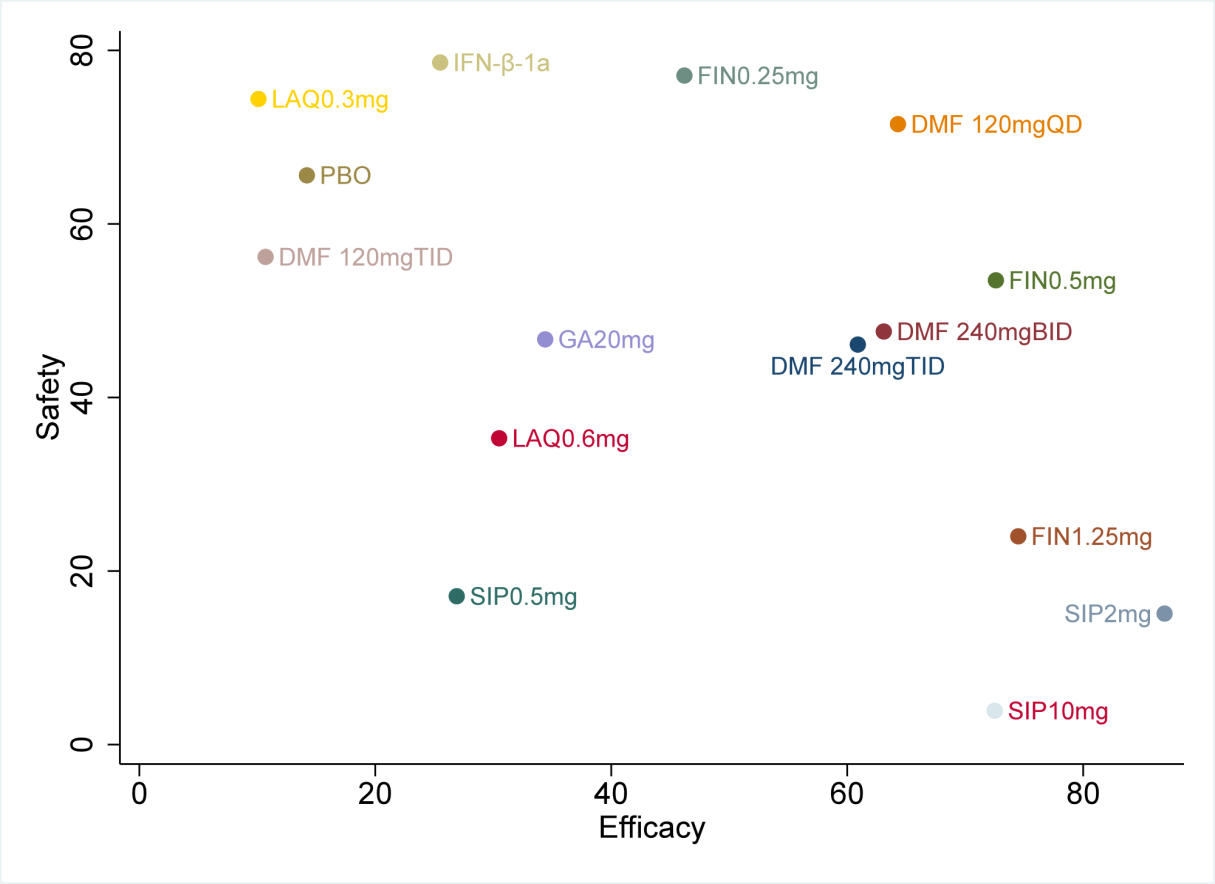


**Supplementary Figure 3:Pairwise Meta‑analysis for AE**


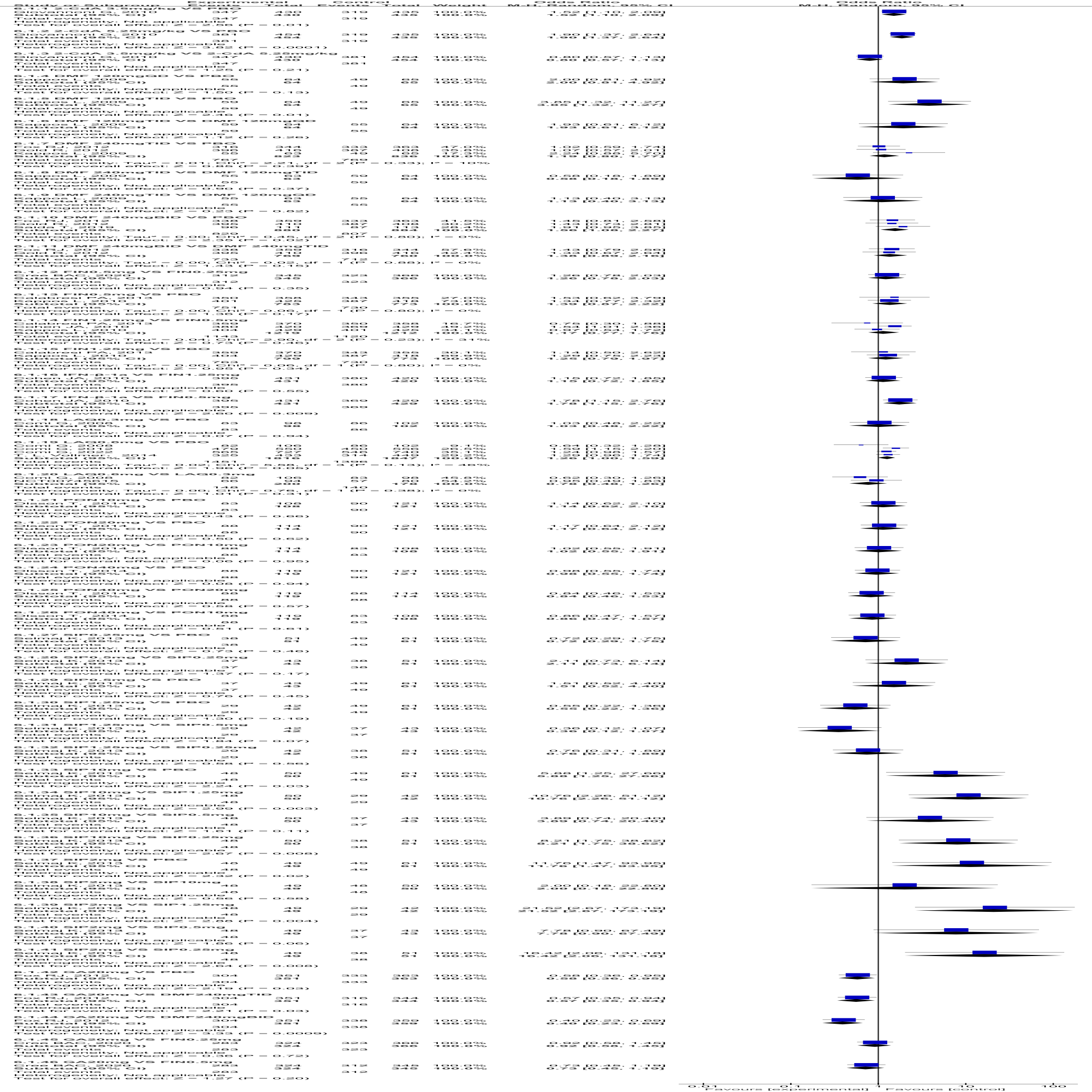


**Supplementary Figure 4:Pairwise Meta‑analysis for SAE**


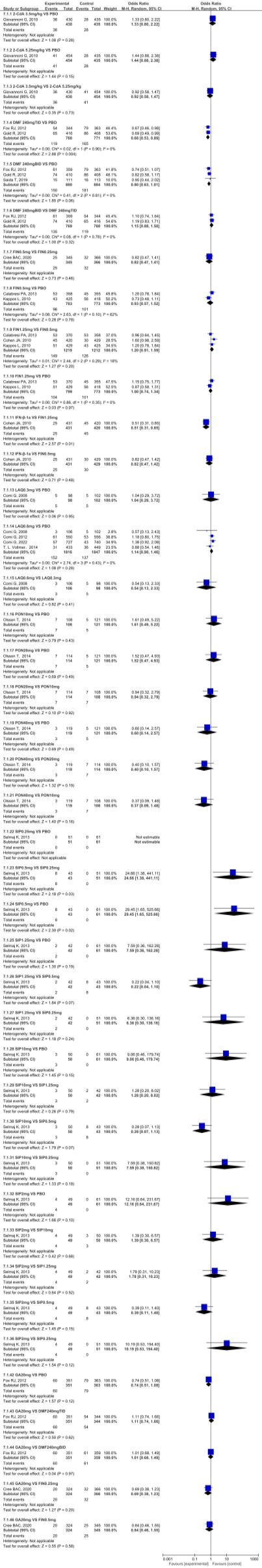


**Supplementary Figure 5:Evidence network diagram for T1**


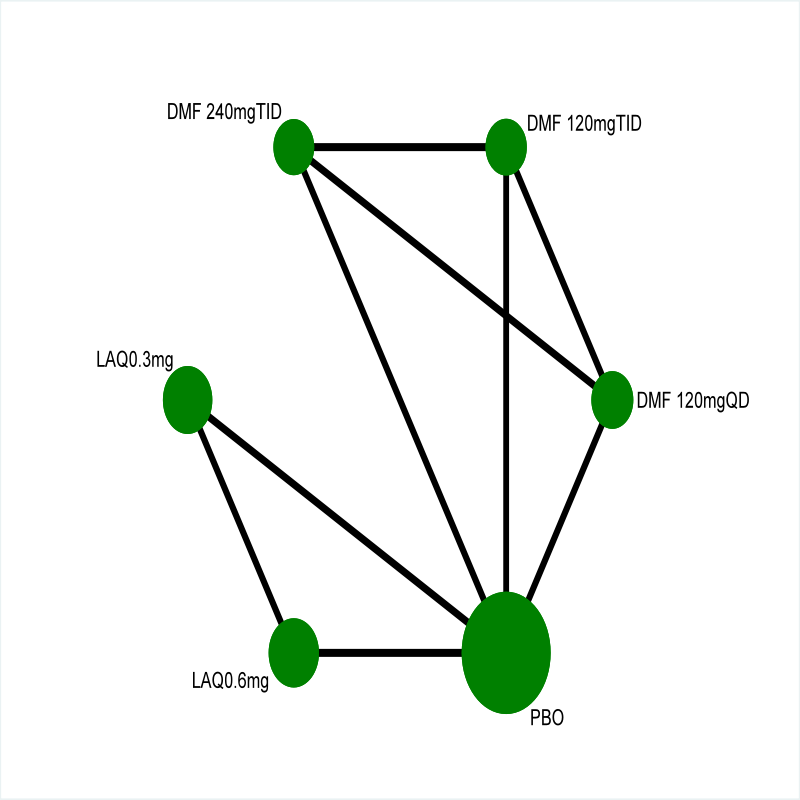


**Supplementary Figure 6:Evidence network diagram for T2**


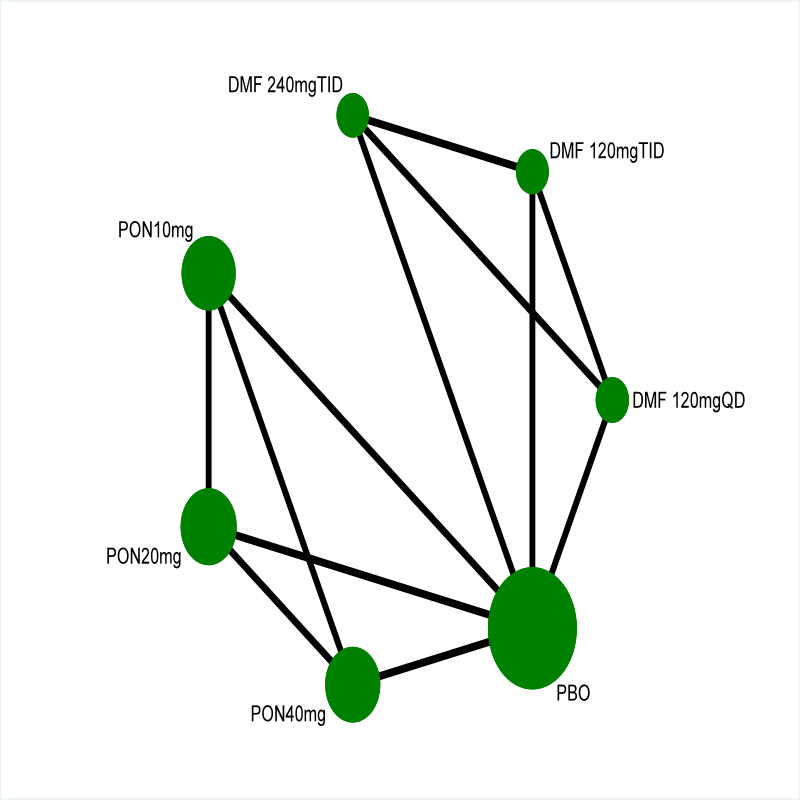


**Supplementary Figure 7:Evidence network diagram for AE**


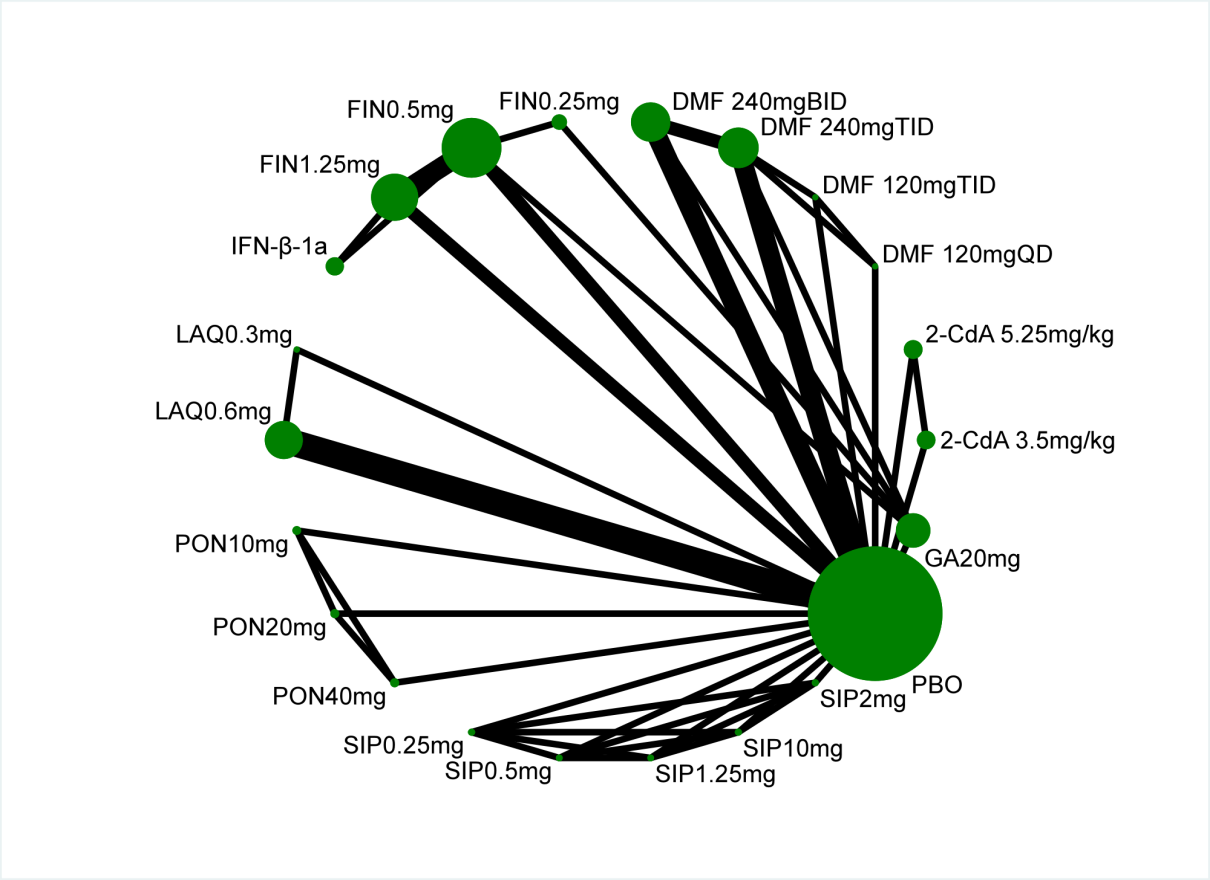


**Supplementary Figure 8:Evidence network diagram for SAE**


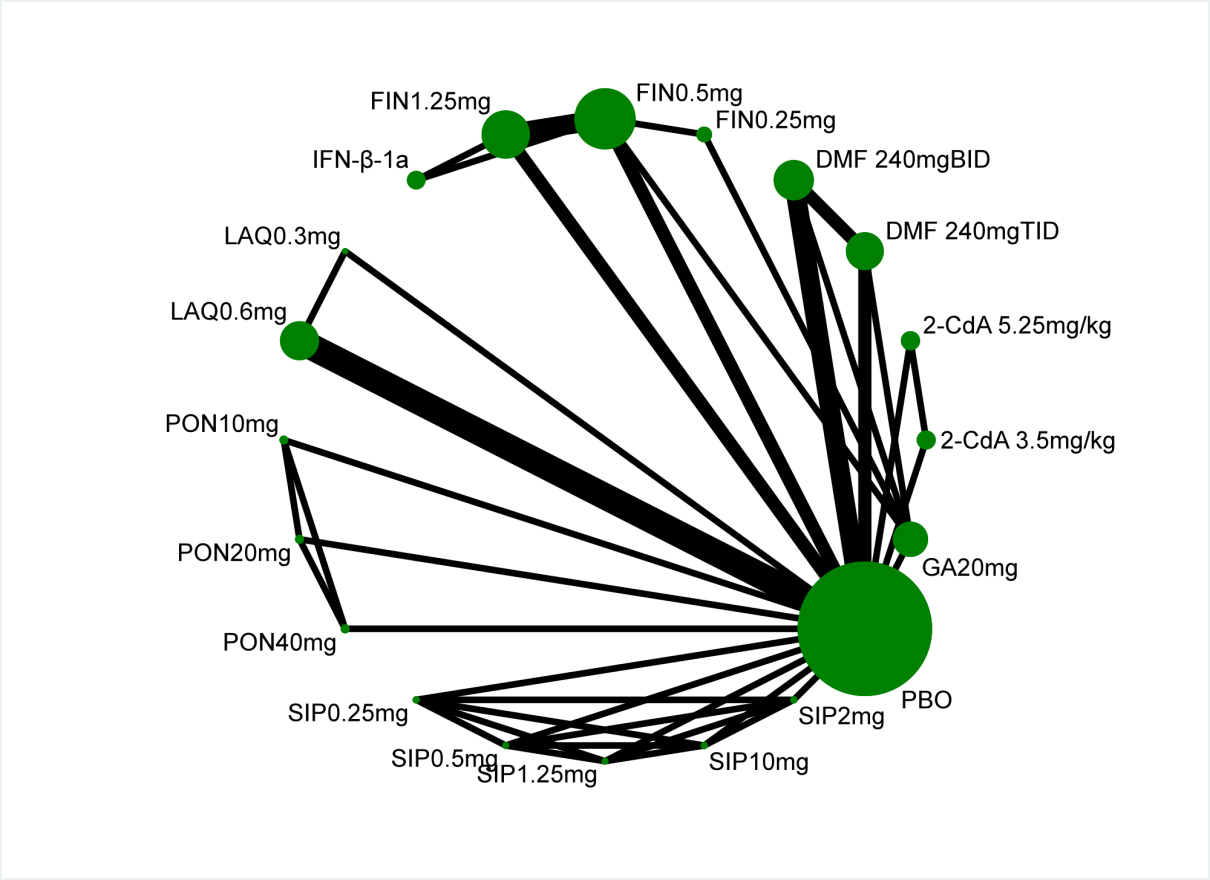


**Supplementary Figure 9:The SUCRA Ranking Diagram of T1**


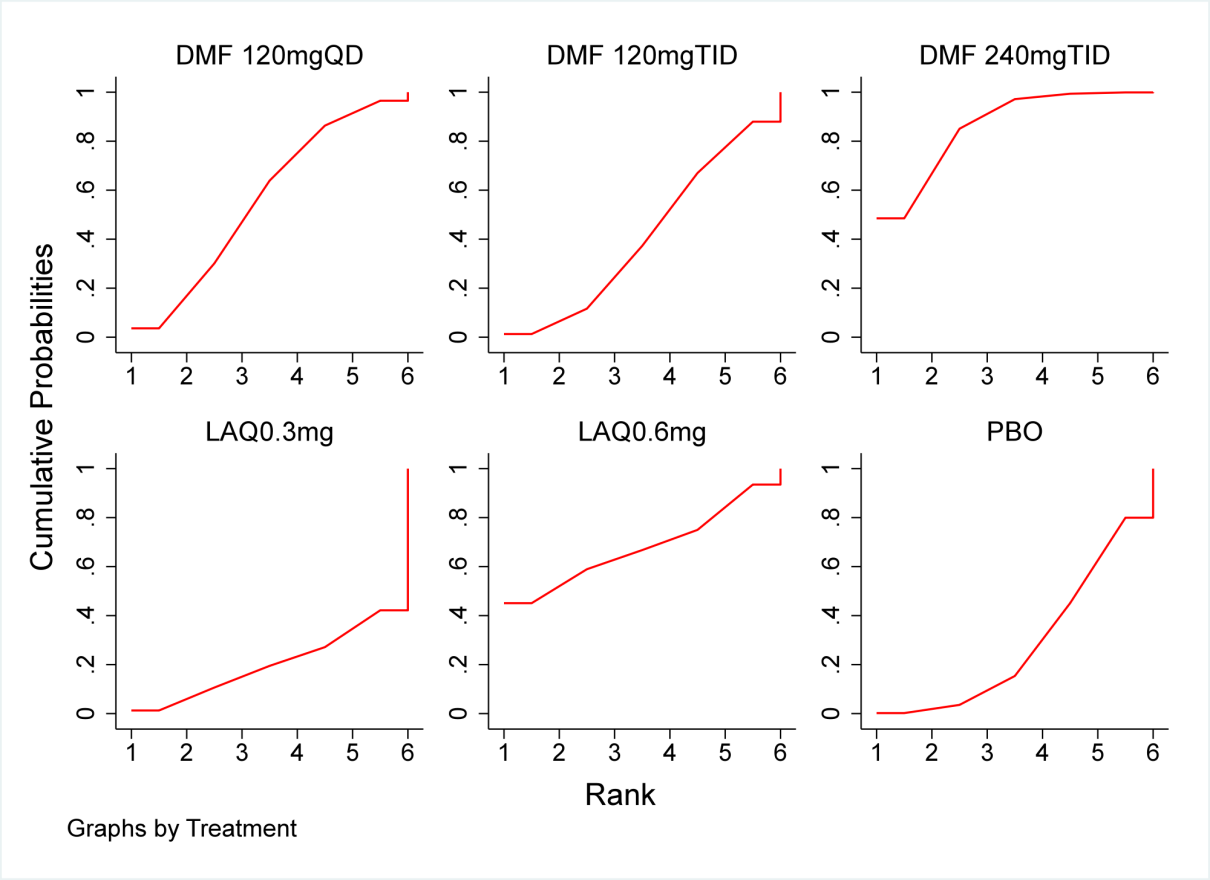


**Supplementary Figure 10:The SUCRA Ranking Diagram of T2**


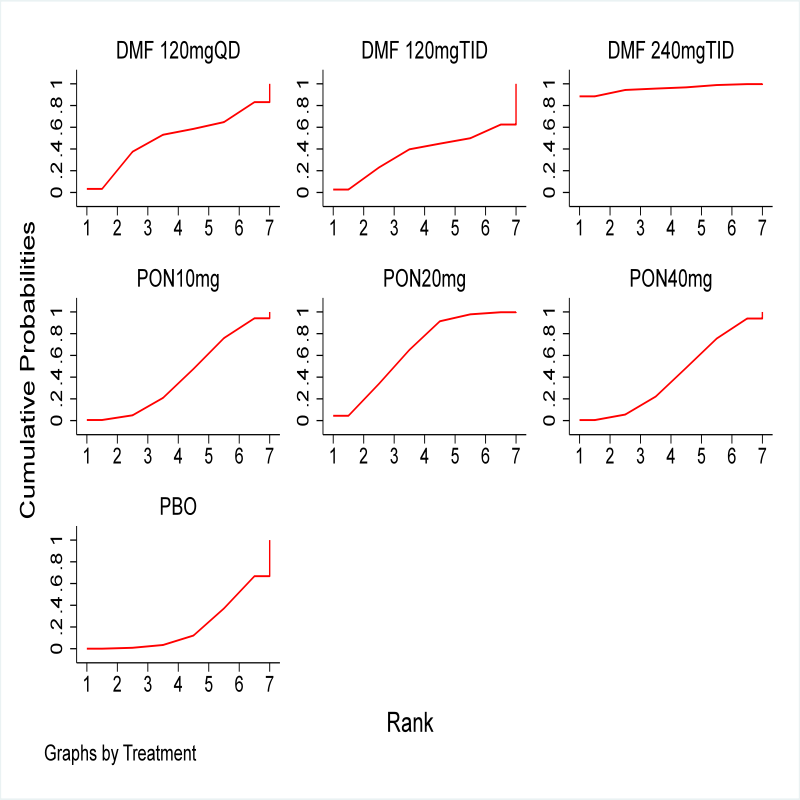


**Supplementary Figure 11:The SUCRA Ranking Diagram of AE**


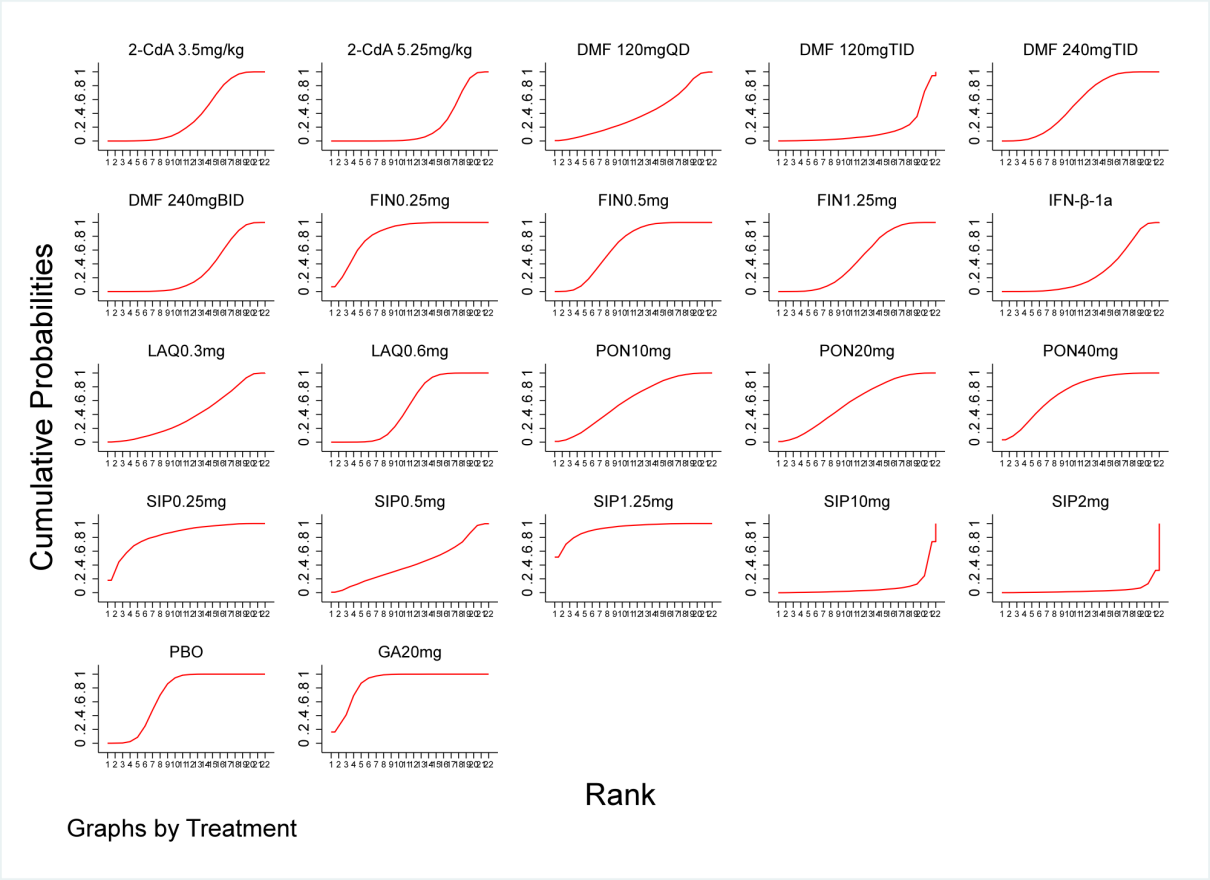


**Supplementary Figure 12:The SUCRA Ranking Diagram of SAE**


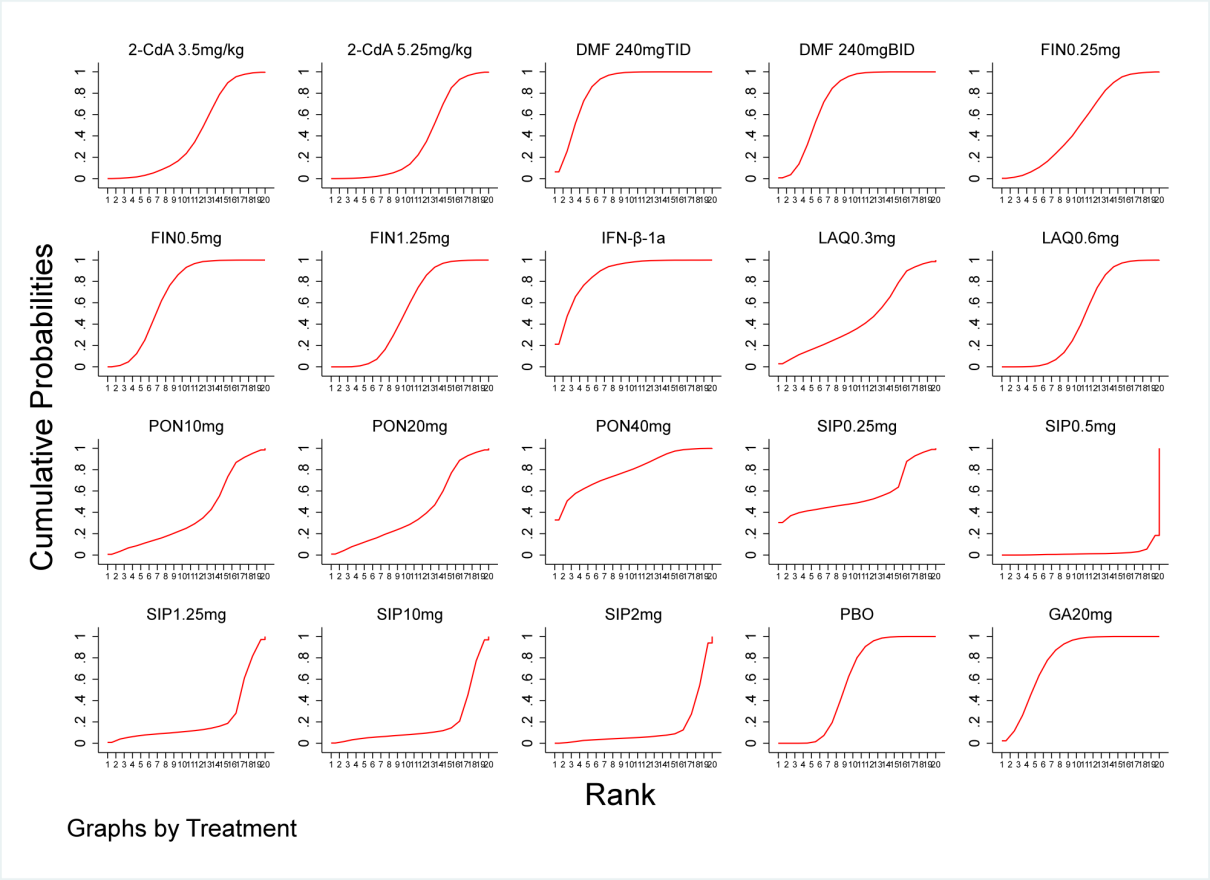


**Supplementary Figure 13:Overall Inconsistency Test of T1**


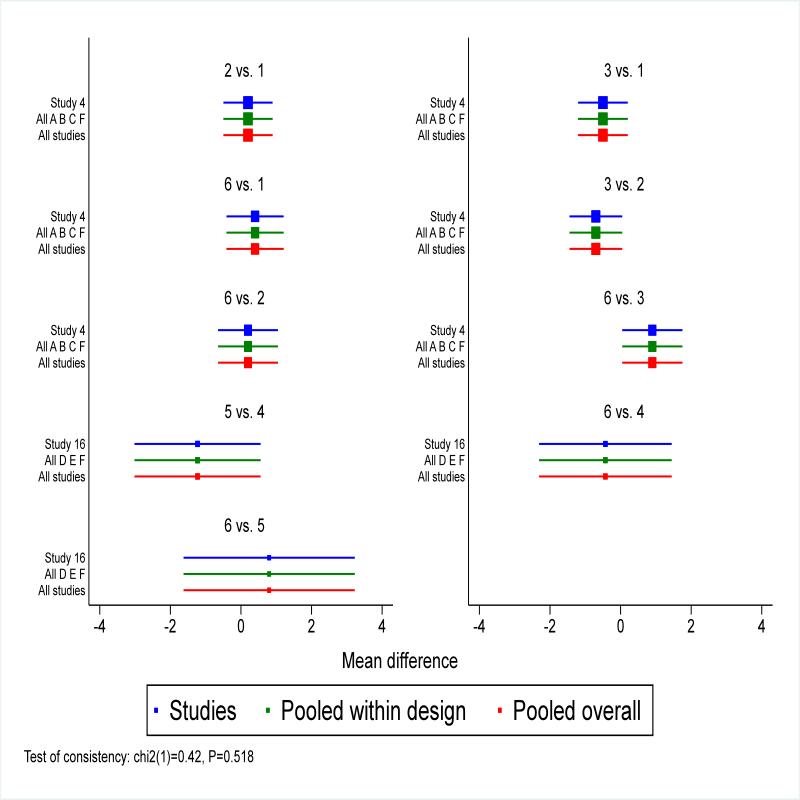


**Supplementary Figure 14:Overall Inconsistency Test of T2**


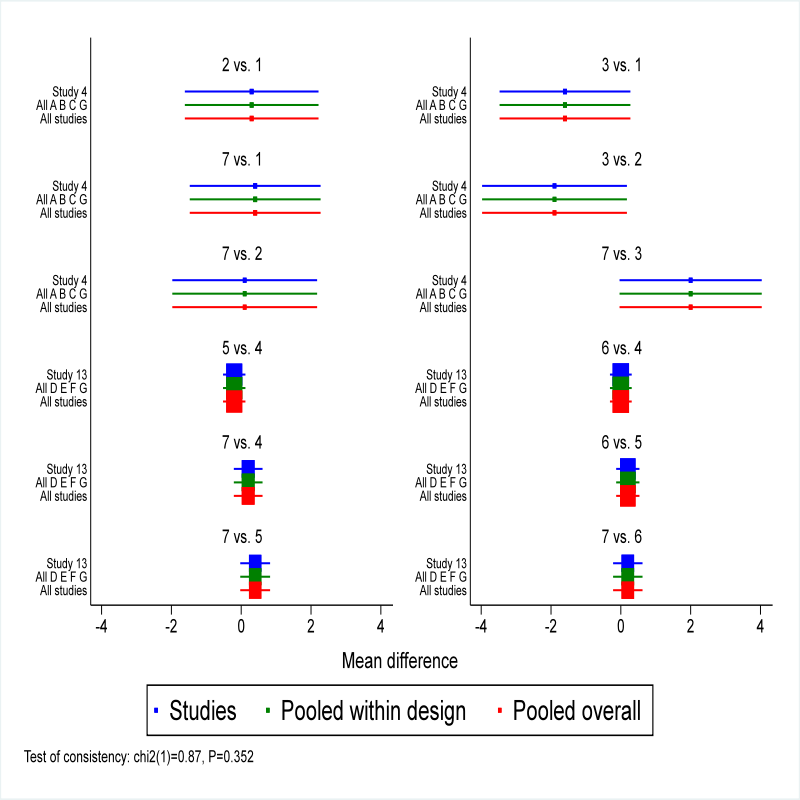


**Supplementary Figure 15:Overall Inconsistency Test of AE**


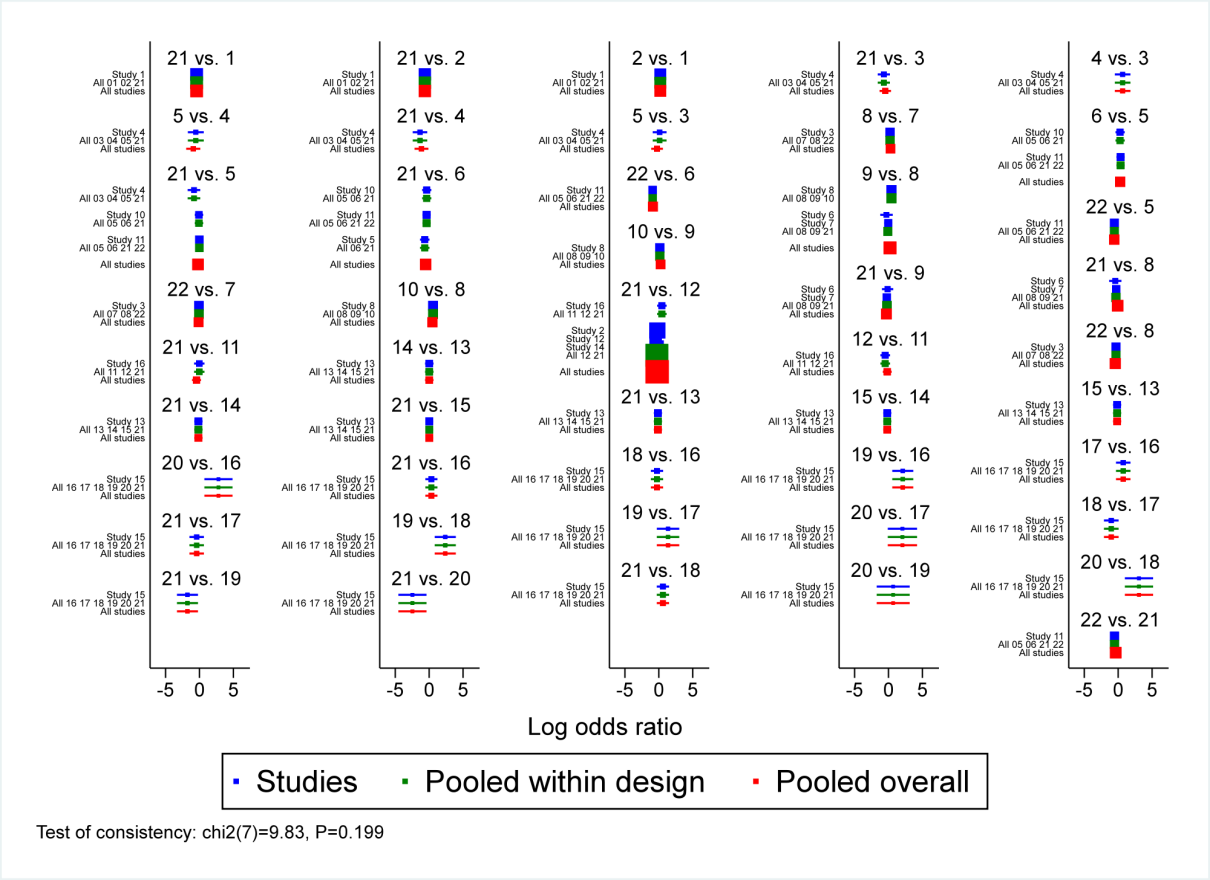


**Supplementary Figure 16:Overall Inconsistency Test of SAE**


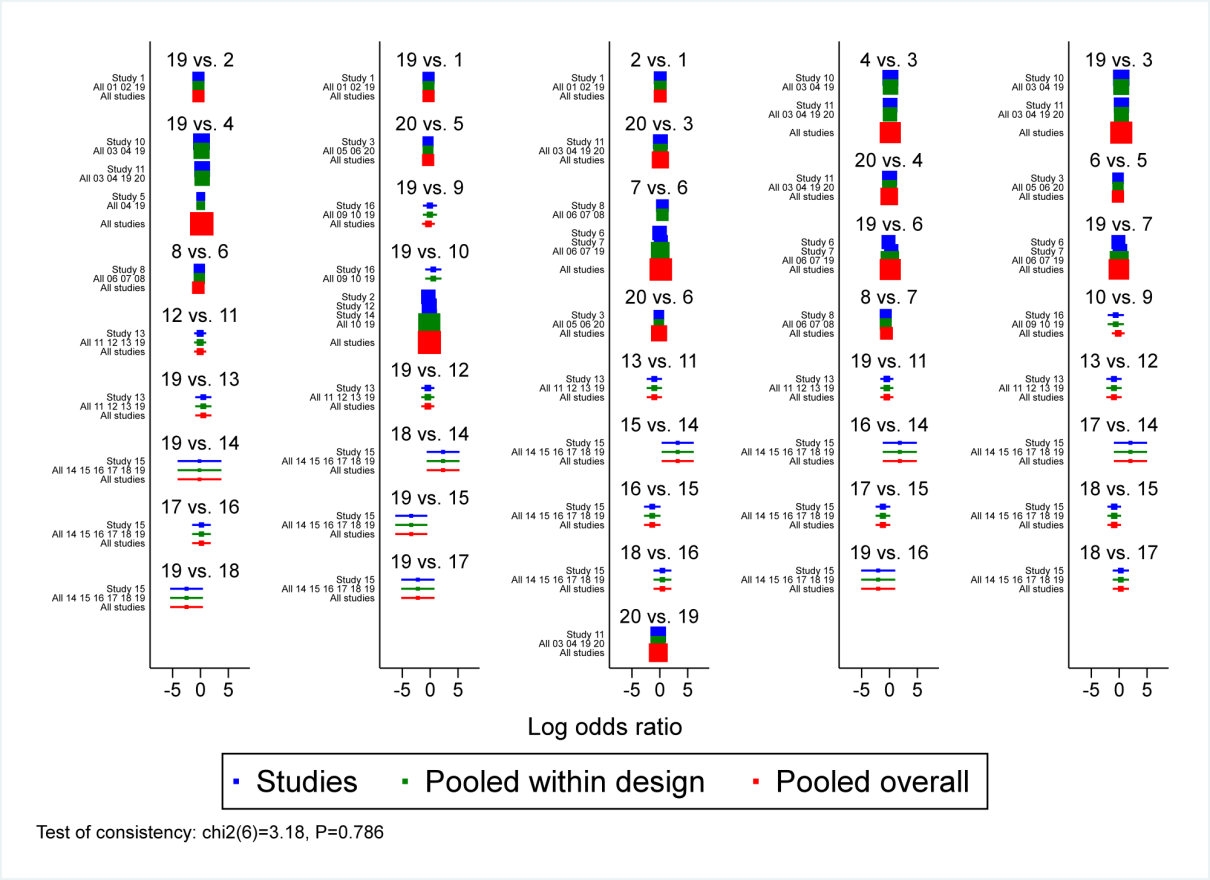


**Supplementary Figure 17:Comparison-adjusted funnel plots of AE**


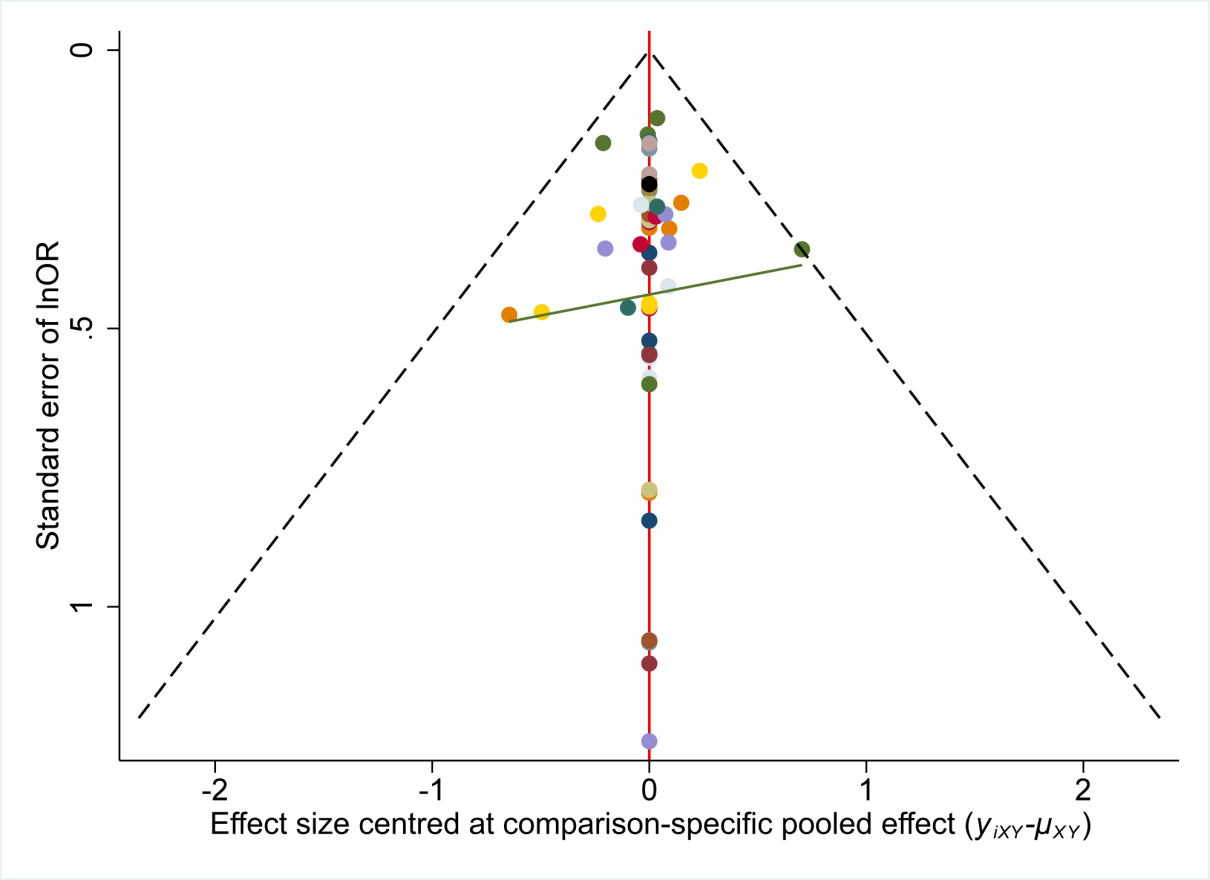


**Supplementary Figure 18:Comparison-adjusted funnel plots of SAE**


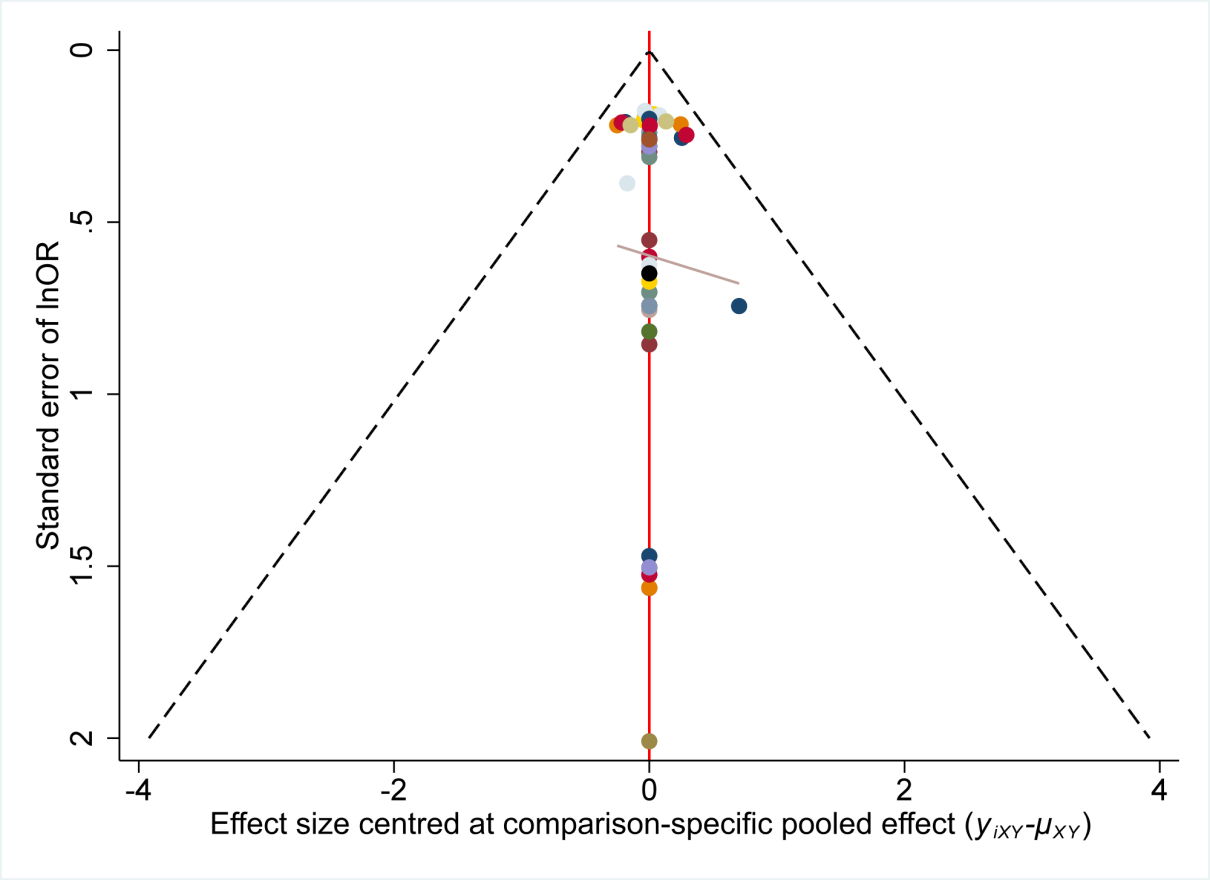

Supplement: Supplementary file 1 [file DataSheet1.docx]
