# Supplementary material for: Efficacy and safety of disease-modifying oral drugs in treatment of relapsing-remitting multiple sclerosis: systematic review and network meta-analysis
Source: Front Immunol. 2026 Mar 16;17:1733948. doi: 10.3389/fimmu.2026.1733948 (PMC13033576; doi:10.3389/fimmu.2026.1733948)
Supplement: Supplementary file 2 [file DataSheet2.docx]

**Supplementary Table 1 Inconsistency Test of ARR**

| Loop | IF | seIF | z_value | p_value | CI_95 | Loop_Heterog_tau2 |
| --- | --- | --- | --- | --- | --- | --- |
| LAQ0.3mg-LAQ0.6mg-PBO | 0.168 | 0.212 | 0.792 | 0.428 | (0.00,0.58) | 0 |
| FIN0.5mg-FIN1.25mg-IFN-β-1a | 0.056 | 0.07 | 0.802 | 0.422 | (0.00,0.19) | 0 |
| FIN0.5mg-FIN1.25mg-PBO | 0.055 | 0.049 | 1.121 | 0.262 | (0.00,0.15) | 0 |
| DMF 240mgTID-DMF 240mgBID-GA20mg | 0.04 | 0.054 | 0.737 | 0.461 | (0.00,0.15) | 0 |
| DMF 240mgTID-DMF 240mgBID-PBO | 0.029 | 0.198 | 0.144 | 0.886 | (0.00,0.42) | 0 |
| DMF 240mgTID-PBO-GA20mg | 0.028 | 0.07 | 0.406 | 0.685 | (0.00,0.16) | 0 |
| DMF 120mgTID-DMF 240mgTID-PBO | 0.027 | 0.26 | 0.104 | 0.918 | (0.00,0.54) | 0 |
| DMF 120mgQD-DMF 240mgTID-PBO | 0.027 | 0.303 | 0.089 | 0.929 | (0.00,0.62) | 0 |
| FIN0.5mg-PBO-GA20mg | 0.017 | 0.066 | 0.258 | 0.796 | (0.00,0.15) | 0 |
| DMF 240mgBID-PBO-GA20mg | 0.01 | 0.07 | 0.142 | 0.887 | (0.00,0.15) | 0 |

**Supplementary Table 2 Inconsistency Test of DAE**

| Loop | IF | seIF | z_value | p_value | CI_95 | Loop_Heterog_tau2 |
| --- | --- | --- | --- | --- | --- | --- |
| FIN0.5mg-PBO-GA20mg | 0.888 | 0.387 | 2.296 | 0.022 | (0.13,1.65) | 0 |
| DMF 240mgTID-DMF 240mgBID-PBO | 0.869 | 1.25 | 0.695 | 0.487 | (0.00,3.32) | 0 |
| FIN0.5mg-FIN1.25mg-IFN-β-1a | 0.302 | 0.471 | 0.641 | 0.522 | (0.00,1.22) | 0 |
| FIN0.5mg-FIN1.25mg-PBO | 0.294 | 0.352 | 0.836 | 0.403 | (0.00,0.98) | 0 |
| DMF 240mgTID-PBO-GA20mg | 0.08 | 0.399 | 0.201 | 0.84 | (0.00,0.86) | 0 |
| DMF 240mgTID-DMF 240mgBID-GA20mg | 0.068 | 0.391 | 0.175 | 0.861 | (0.00,0.83) | 0 |
| DMF 240mgBID-PBO-GA20mg | 0.01 | 0.396 | 0.025 | 0.98 | (0.00,0.79) | 0 |

**Supplementary Table 3** **Network Inconsistency Test of ARR**

| Side | Direct | | Indirect | | Difference | | P>z |
| --- | --- | --- | --- | --- | --- | --- | --- |
|  | Coef. | Std. Err. | Coef. | Std. Err. | Coef. | Std. Err. |  |
| 2-CdA3.5mg/kg-2-CdA5.25mg/kg | 0.0100038 | 0.0181171 | -0.1106539 | 133.2415 | 0.1206577 | 133.2415 | 0.999 |
| 2-CdA 3.5mg/kg-PBO | 0.19 | 0.0263741 | 0.2767884 | 46.04639 | -0.0867884 | 46.04639 | 0.998 |
| 2-CdA 5.25mg/kg-PBO | 0.1799998 | 0.0262126 | 0.0663121 | 191.7584 | 0.1136877 | 191.7584 | 1 |
| DMF 120mgQD-DMF 240mgTID | 0.02 | 0.1749059 | 0.0762984 | 0.3477765 | -0.0562984 | 0.3938588 | 0.886 |
| DMF 120mgQD-PBO | 0.23 | 0.1902266 | 0.1737016 | 0.322748 | 0.0562984 | 0.3938588 | 0.886 |
| DMF 120mgTID-DMF 240mgTID | -0.34 | 0.2063936 | -0.2837016 | 0.3646296 | -0.0562984 | 0.3938588 | 0.886 |
| DMF 120mgTID-PBO | -0.13 | 0.2195277 | -0.1862984 | 0.3408409 | 0.0562984 | 0.3938588 | 0.886 |
| DMF 240mgTID-DMF 240mgBID | -0.0043522 | 0.0211345 | 0.0519461 | 0.3932854 | -0.0562984 | 0.3938592 | 0.886 |
| DMF 240mgTID-PBO | 0.1839564 | 0.0304782 | 0.1688129 | 0.1104271 | 0.0151435 | 0.1187479 | 0.899 |
| DMF 240mgTID-GA20mg | 0.0901494 | 0.0322419 | 0.0528672 | 0.0524109 | 0.0372822 | 0.0614446 | 0.544 |
| DMF 240mgBID-PBO | 0.1876195 | 0.0306373 | 0.1781034 | 0.1073277 | 0.0095162 | 0.1156897 | 0.934 |
| DMF 240mgBID-GA20mg | 0.0706173 | 0.0341753 | 0.1137643 | 0.0510657 | -0.043147 | 0.062072 | 0.487 |
| FIN0.25mg-FIN0.5mg | -0.07 | 0.0398921 | -0.0548565 | 0.1145918 | -0.0151435 | 0.1187479 | 0.899 |
| FIN0.25mg-GA20mg | 0.04 | 0.0434135 | 0.0248565 | 0.1106852 | 0.0151435 | 0.1187479 | 0.899 |
| FIN0.5mg-FIN1.25mg | -0.0038915 | 0.0161621 | 0.011252 | 0.1174862 | -0.0151435 | 0.1187478 | 0.899 |
| FIN0.5mg-IFN-β-1a | 0.17 | 0.0470468 | 0.058726 | 0.0780635 | 0.111274 | 0.0776451 | 0.152 |
| FIN0.5mg-PBO | 0.1984818 | 0.0244336 | 0.2480188 | 0.0443739 | -0.049537 | 0.0490318 | 0.312 |
| FIN0.5mg-GA20mg | 0.11 | 0.0396728 | 0.1024282 | 0.0441739 | 0.0075718 | 0.0593739 | 0.899 |
| FIN1.25mg-IFN-β-1a | 0.13 | 0.0481679 | 0.241274 | 0.0759847 | -0.111274 | 0.0776451 | 0.152 |
| FIN1.25mg-PBO | 0.2210432 | 0.0238078 | 0.1524921 | 0.0595911 | 0.0685511 | 0.0627974 | 0.275 |
| LAQ0.3mg-LAQ0.6mg | -0.24 | 0.1363863 | 0.0961005 | 0.2859917 | -0.3361005 | 0.3084309 | 0.276 |
| LAQ0.3mg-PBO | 0.01 | 0.1610433 | -0.3261003 | 0.2445201 | 0.3361004 | 0.3084309 | 0.276 |
| LAQ0.6mg-PBO | 0.0853336 | 0.0216619 | 0.3850952 | 208.9655 | -0.2997616 | 208.9655 | 0.999 |
| PON10mg-PON40mg | -0.0013909 | 2.502918 | -0.0007946 | 370.2859 | -0.0005964 | 370.2969 | 0.999 |
| PON10mg-PBO | 0.1929998 | 0.1395605 | 0.1816178 | 703.6353 | 0.0113821 | 703.6353 | 0.999 |
| PON20mg-PBO | 0.108 | 0.1441933 | 0.120035 | 703.7511 | -0.012035 | 703.7511 | 0.999 |
| PON40mg-PBO | 0.274 | 0.1311186 | 0.3848882 | 601.1528 | -0.1108882 | 601.1528 | 0.999 |
| SIP0.5mg-SIP2mg | -0.4099959 | 0.3073604 | 1.632091 | 2348.64 | -2.042087 | 2348.64 | 0.999 |
| SIP0.5mg-PBO | -0.03 | 0.2455861 | 0.3791543 | 616.524 | -0.4091543 | 616.5241 | 0.999 |
| SIP10mg-PBO | 0.28 | 0.2012511 | 0.7977271 | 636.6163 | -0.5177271 | 636.6163 | 0.999 |
| SIP2mg-PBO | 0.38 | 0.1940818 | 0.8472938 | 656.7846 | -0.4672938 | 656.7847 | 0.999 |
| PBO-GA20mg | -0.1093616 | 0.0456695 | -0.0966641 | 0.0416858 | -0.0126974 | 0.0653385 | 0.846 |

**Supplementary Table 4 Network Inconsistency Test of DAE**

| Side | Direct | | Indirect | | Difference | | P>z |
| --- | --- | --- | --- | --- | --- | --- | --- |
|  | Coef. | Std. Err. | Coef. | Std. Err. | Coef. | Std. Err. |  |
| DMF 120mgQD-DMF 120mgTID | 0.3709584 | 0.6142883 | -0.2791493 | 194.404 | 0.6501077 | 194.4052 | 0.997 |
| DMF 120mgQD-DMF 240mgTID | 0.5402079 | 0.6001091 | 0.2146069 | 93.99946 | 0.325601 | 94.0014 | 0.997 |
| DMF 120mgTID-DMF 240mgTID | 0.1692495 | 0.5509835 | -0.4833629 | 187.0403 | 0.6526124 | 187.041 | 0.997 |
| DMF 240mgTID-DMF 240mgBID | -0.008784 | 0.1465622 | -1.818689 | 2.477518 | 1.809905 | 2.481816 | 0.466 |
| DMF 240mgTID-PBO | -0.1941164 | 0.151721 | -0.9340034 | 0.7674316 | 0.739887 | 0.7835881 | 0.345 |
| DMF 240mgTID-GA20mg | -0.1912635 | 0.2436498 | 0.0270612 | 0.3416258 | -0.2183248 | 0.4160667 | 0.6 |
| DMF 240mgBID-PBO | -0.1719103 | 0.1504304 | -1.177893 | 0.8035183 | 1.005983 | 0.8192594 | 0.219 |
| DMF 240mgBID-GA20mg | -0.2222023 | 0.2398125 | 0.1435752 | 0.3419835 | -0.3657775 | 0.4125492 | 0.375 |
| FIN0.25mg-FIN0.5mg | 0.2496958 | 0.2728218 | 1.255679 | 0.7796432 | -1.005983 | 0.8192627 | 0.219 |
| FIN0.25mg-GA20mg | 0.705614 | 0.2565021 | -0.3003711 | 0.7960895 | 1.005985 | 0.8192626 | 0.219 |
| FIN0.5mg-FIN1.25mg | 0.6756266 | 0.173899 | 1.68161 | 0.7900406 | -1.005983 | 0.8192627 | 0.219 |
| FIN0.5mg-IFN-β-1a | -0.4298564 | 0.3302203 | -0.0819158 | 0.632996 | -0.3479406 | 0.6842379 | 0.611 |
| FIN0.5mg-PBO | 0.0180278 | 0.2600677 | 0.3667599 | 0.295078 | -0.3487321 | 0.3933032 | 0.375 |
| FIN0.5mg-GA20mg | 0.455918 | 0.2454615 | -0.0470739 | 0.3279429 | 0.5029919 | 0.4096314 | 0.219 |
| FIN1.25mg-IFN-β-1a | -1.058465 | 0.3022659 | -1.406407 | 0.6735922 | 0.3479414 | 0.6842382 | 0.611 |
| FIN1.25mg-PBO | -0.6769583 | 0.2288277 | -0.1721469 | 0.4194135 | -0.5048115 | 0.4745137 | 0.287 |
| LAQ0.3mg-LAQ0.6mg | -0.0800457 | 1.009965 | 2.933191 | 1.420243 | -3.013236 | 1.742276 | 0.084 |
| LAQ0.3mg-PBO | 0.9059326 | 0.8489543 | -2.107296 | 1.707334 | 3.013229 | 1.742273 | 0.084 |
| LAQ0.6mg-PBO | -0.4433069 | 0.1922434 | 0.6135122 | 313.9668 | -1.056819 | 313.9669 | 0.997 |
| SIP0.25mg-PBO | 0.5276328 | 1.239738 | 0.1001862 | 637.5297 | 0.4274466 | 637.5333 | 0.999 |
| SIP0.5mg-PBO | -1.356242 | 0.8621281 | -1.815607 | 630.1478 | 0.4593652 | 630.1488 | 0.999 |
| SIP1.25mg-PBO | 0.3291818 | 1.241507 | -0.0892874 | 638.6247 | 0.4184692 | 638.6251 | 0.999 |
| SIP10mg-PBO | -1.998096 | 0.8012173 | -2.413687 | 631.0499 | 0.4155915 | 631.0511 | 0.999 |
| SIP2mg-PBO | -1.41495 | 0.8407566 | -1.83519 | 631.5319 | 0.4202403 | 631.5329 | 0.999 |
| PBO-GA20mg | -0.0510997 | 0.2472105 | 0.3057613 | 0.2802288 | -0.356861 | 0.3715599 | 0.337 |

**Supplementary Table 5:Network Meta-analysis for T1 (Mean Difference, 95% CI)**

| DMF 240mgTID |  |  |  |  |  |
| --- | --- | --- | --- | --- | --- |
| -0.10 (-2.67,2.47) | LAQ0.6mg |  |  |  |  |
| -0.50 (-1.21,0.21) | -0.40 (-2.96,2.16) | DMF 120mgQD |  |  |  |
| -0.70 (-1.45,0.05) | -0.60 (-3.17,1.97) | -0.20 (-0.90,0.50) | DMF 120mgTID |  |  |
| **-0.90 (-1.75,-0.05)** | -0.80 (-3.23,1.63) | -0.40 (-1.21,0.41) | -0.20 (-1.05,0.65) | PBO |  |
| -1.33 (-3.39,0.73) | -1.23 (-3.02,0.56) | -0.83 (-2.88,1.22) | -0.63 (-2.69,1.43) | -0.43 (-2.31,1.45) | LAQ0.3mg |

Note: Bold text indicates data with statistically significant differences.

**Supplementary Table 6:Network Meta-analysis for T2 (Mean Difference, 95% CI)**

| DMF 240mgTID |  |  |  |  |  |  |
| --- | --- | --- | --- | --- | --- | --- |
| -1.60 (-3.68,0.48) | PON20mg |  |  |  |  |  |
| -1.60 (-3.47,0.27) | -0.00 (-1.92,1.92) | DMF 120mgQD |  |  |  |  |
| -1.80 (-3.88,0.28) | -0.20 (-0.52,0.12) | -0.20 (-2.12,1.72) | PON10mg |  |  |  |
| -1.80 (-3.88,0.28) | -0.20 (-0.53,0.13) | -0.20 (-2.12,1.72) | -0.00 (-0.31,0.31) | PON40mg |  |  |
| -1.90 (-3.97,0.17) | -0.30 (-2.42,1.82) | -0.30 (-2.21,1.61) | -0.10 (-2.21,2.01) | -0.10 (-2.22,2.02) | DMF 120mgTID |  |
| -2.00 (-4.04,0.04) | -0.40 (-0.83,0.03) | -0.40 (-2.27,1.47) | -0.20 (-0.61,0.21) | -0.20 (-0.62,0.22) | -0.10 (-2.17,1.97) | PBO |

Note: Bold text indicates data with statistically significant differences.

**Supplementary Table 7:Network Meta-analysis for AE (Odds ratio, 95% CI)**

| SIP1.25mg |  |  |  |  |  |  |  |  |  |  |  |  |  |  |  |  |  |  |  |  |  |
| --- | --- | --- | --- | --- | --- | --- | --- | --- | --- | --- | --- | --- | --- | --- | --- | --- | --- | --- | --- | --- | --- |
| 0.78 (0.29,2.08) | GA20mg |  |  |  |  |  |  |  |  |  |  |  |  |  |  |  |  |  |  |  |  |
| 0.76 (0.31,1.89) | 0.98 (0.37,2.55) | SIP0.25mg |  |  |  |  |  |  |  |  |  |  |  |  |  |  |  |  |  |  |  |
| 0.68 (0.24,1.93) | 0.87 (0.56,1.35) | 0.89 (0.32,2.48) | FIN0.25mg |  |  |  |  |  |  |  |  |  |  |  |  |  |  |  |  |  |  |
| 0.55 (0.22,1.36) | **0.70 (0.49,1.00)** | 0.72 (0.29,1.75) | 0.80 (0.49,1.32) | PBO |  |  |  |  |  |  |  |  |  |  |  |  |  |  |  |  |  |
| 0.56 (0.19,1.64) | 0.71 (0.36,1.41) | 0.73 (0.25,2.12) | 0.82 (0.38,1.76) | 1.02 (0.57,1.82) | PON40mg |  |  |  |  |  |  |  |  |  |  |  |  |  |  |  |  |
| 0.51 (0.19,1.35) | **0.65 (0.44,0.95)** | 0.67 (0.25,1.74) | 0.75 (0.48,1.17) | 0.93 (0.65,1.33) | 0.91 (0.46,1.79) | FIN0.5mg |  |  |  |  |  |  |  |  |  |  |  |  |  |  |  |
| 0.48 (0.16,1.42) | 0.61 (0.30,1.23) | 0.63 (0.21,1.84) | 0.70 (0.32,1.54) | 0.87 (0.48,1.60) | 0.86 (0.47,1.57) | 0.94 (0.47,1.90) | PON10mg |  |  |  |  |  |  |  |  |  |  |  |  |  |  |
| 0.47 (0.16,1.39) | 0.60 (0.30,1.20) | 0.61 (0.21,1.80) | 0.69 (0.31,1.50) | 0.86 (0.47,1.56) | 0.84 (0.46,1.53) | 0.92 (0.46,1.85) | 0.98 (0.52,1.83) | PON20mg |  |  |  |  |  |  |  |  |  |  |  |  |  |
| 0.44 (0.17,1.17) | **0.56 (0.37,0.85)** | 0.58 (0.22,1.51) | 0.65 (0.37,1.13) | 0.81 (0.56,1.15) | 0.79 (0.40,1.55) | 0.87 (0.54,1.38) | 0.92 (0.46,1.86) | 0.94 (0.47,1.89) | DMF 240mgTID |  |  |  |  |  |  |  |  |  |  |  |  |
| 0.43 (0.17,1.07) | **0.54 (0.37,0.80)** | 0.56 (0.23,1.38) | 0.62 (0.37,1.06) | **0.78 (0.66,0.91)** | 0.76 (0.42,1.39) | 0.84 (0.57,1.24) | 0.89 (0.48,1.66) | 0.91 (0.49,1.68) | 0.97 (0.65,1.43) | LAQ0.6mg |  |  |  |  |  |  |  |  |  |  |  |
| 0.40 (0.15,1.09) | **0.52 (0.33,0.81)** | 0.53 (0.20,1.40) | **0.59 (0.35,1.00)** | 0.74 (0.50,1.09) | 0.72 (0.36,1.45) | 0.80 (0.58,1.09) | 0.85 (0.41,1.74) | 0.86 (0.42,1.76) | 0.92 (0.56,1.52) | 0.95 (0.63,1.45) | FIN1.25mg |  |  |  |  |  |  |  |  |  |  |
| 0.36 (0.12,1.07) | 0.46 (0.15,1.43) | 0.47 (0.16,1.38) | 0.53 (0.16,1.73) | 0.66 (0.23,1.93) | 0.65 (0.19,2.18) | 0.71 (0.23,2.20) | 0.76 (0.22,2.59) | 0.77 (0.23,2.63) | 0.82 (0.27,2.54) | 0.85 (0.29,2.51) | 0.89 (0.29,2.79) | SIP0.5mg |  |  |  |  |  |  |  |  |  |
| 0.35 (0.12,1.08) | **0.45 (0.21,0.95)** | 0.46 (0.15,1.40) | 0.52 (0.23,1.18) | 0.65 (0.34,1.25) | 0.63 (0.26,1.51) | 0.69 (0.33,1.47) | 0.74 (0.30,1.80) | 0.75 (0.31,1.83) | 0.80 (0.38,1.69) | 0.83 (0.43,1.60) | 0.87 (0.41,1.87) | 0.98 (0.28,3.42) | LAQ0.3mg |  |  |  |  |  |  |  |  |
| 0.34 (0.10,1.18) | 0.44 (0.18,1.07) | 0.45 (0.13,1.52) | 0.50 (0.19,1.32) | 0.62 (0.27,1.45) | 0.61 (0.22,1.70) | 0.67 (0.27,1.66) | 0.71 (0.25,2.02) | 0.73 (0.26,2.05) | 0.77 (0.33,1.84) | 0.80 (0.34,1.89) | 0.84 (0.33,2.12) | 0.94 (0.24,3.68) | 0.96 (0.33,2.81) | DMF 120mgQD |  |  |  |  |  |  |  |
| **0.36 (0.14,0.94)** | **0.46 (0.28,0.74)** | 0.47 (0.18,1.21) | **0.53 (0.29,0.96)** | **0.66 (0.48,0.91)** | 0.64 (0.33,1.25) | 0.71 (0.44,1.14) | 0.75 (0.38,1.49) | 0.77 (0.39,1.51) | 0.82 (0.51,1.32) | 0.85 (0.59,1.21) | 0.89 (0.54,1.47) | 0.99 (0.33,3.03) | 1.02 (0.49,2.11) | 1.06 (0.43,2.61) | 2-CdA 3.5mg/kg |  |  |  |  |  |  |
| **0.33 (0.12,0.87)** | **0.42 (0.27,0.64)** | 0.43 (0.16,1.12) | **0.48 (0.27,0.85)** | **0.60 (0.42,0.86)** | 0.58 (0.30,1.16) | 0.64 (0.40,1.03) | 0.68 (0.34,1.38) | 0.70 (0.35,1.40) | 0.74 (0.49,1.12) | 0.77 (0.52,1.14) | 0.81 (0.49,1.34) | 0.90 (0.29,2.79) | 0.93 (0.44,1.96) | 0.96 (0.39,2.37) | 0.91 (0.56,1.47) | DMF 240mgBID |  |  |  |  |  |
| **0.31 (0.11,0.89)** | **0.40 (0.23,0.69)** | 0.41 (0.14,1.14) | **0.46 (0.25,0.84)** | **0.57 (0.34,0.96)** | 0.56 (0.25,1.21) | **0.61 (0.40,0.93)** | 0.65 (0.29,1.44) | 0.66 (0.30,1.47) | 0.70 (0.38,1.29) | 0.73 (0.42,1.26) | 0.77 (0.49,1.19) | 0.86 (0.26,2.82) | 0.88 (0.38,2.03) | 0.91 (0.34,2.45) | 0.86 (0.47,1.59) | 0.95 (0.51,1.75) | IFN-β-1a |  |  |  |  |
| **0.29 (0.11,0.76)** | **0.37 (0.23,0.60)** | **0.38 (0.15,0.98)** | **0.42 (0.23,0.77)** | **0.53 (0.38,0.73)** | **0.52 (0.26,1.00)** | **0.57 (0.35,0.92)** | 0.60 (0.30,1.20) | 0.61 (0.31,1.22) | 0.65 (0.40,1.06) | **0.68 (0.47,0.97)** | 0.71 (0.43,1.18) | 0.80 (0.26,2.43) | 0.82 (0.39,1.70) | 0.85 (0.34,2.10) | 0.80 (0.57,1.13) | 0.88 (0.54,1.43) | 0.93 (0.50,1.72) | 2-CdA 5.25mg/kg |  |  |  |
| **0.18 (0.04,0.69)** | **0.23 (0.08,0.66)** | **0.23 (0.06,0.90)** | **0.26 (0.08,0.80)** | **0.32 (0.12,0.90)** | 0.32 (0.10,1.02) | 0.35 (0.12,1.02) | 0.37 (0.11,1.21) | 0.38 (0.11,1.23) | 0.40 (0.14,1.13) | 0.41 (0.15,1.17) | 0.44 (0.15,1.30) | 0.49 (0.11,2.14) | 0.50 (0.15,1.69) | 0.52 (0.16,1.64) | 0.49 (0.17,1.44) | 0.54 (0.18,1.58) | 0.57 (0.18,1.79) | 0.61 (0.21,1.80) | DMF 120mgTID |  |  |
| **0.09 (0.02,0.44)** | **0.12 (0.02,0.58)** | **0.12 (0.03,0.57)** | **0.14 (0.03,0.69)** | **0.17 (0.04,0.80)** | **0.17 (0.03,0.87)** | **0.18 (0.04,0.90)** | 0.19 (0.04,1.03) | 0.20 (0.04,1.04) | 0.21 (0.04,1.03) | 0.22 (0.05,1.04) | 0.23 (0.05,1.13) | 0.26 (0.05,1.35) | 0.26 (0.05,1.42) | 0.27 (0.05,1.60) | 0.26 (0.05,1.26) | 0.28 (0.06,1.40) | 0.30 (0.06,1.54) | 0.32 (0.07,1.57) | 0.53 (0.08,3.38) | SIP10mg |  |
| **0.05 (0.01,0.37)** | **0.06 (0.01,0.49)** | **0.06 (0.01,0.49)** | **0.07 (0.01,0.58)** | **0.09 (0.01,0.68)** | **0.08 (0.01,0.72)** | **0.09 (0.01,0.75)** | **0.10 (0.01,0.85)** | **0.10 (0.01,0.86)** | **0.11 (0.01,0.87)** | **0.11 (0.01,0.88)** | **0.11 (0.01,0.95)** | 0.13 (0.01,1.11) | 0.13 (0.01,1.16) | 0.14 (0.01,1.29) | 0.13 (0.02,1.06) | 0.14 (0.02,1.17) | 0.15 (0.02,1.28) | 0.16 (0.02,1.32) | 0.26 (0.03,2.68) | 0.50 (0.04,5.70) | SIP2mg |

Note: Bold text indicates data with statistically significant differences.

**Supplementary Table 8:Network Meta-analysis for SAE (Odds ratio, 95% CI)**

| IFN-β-1a |  |  |  |  |  |  |  |  |  |  |  |  |  |  |  |  |  |  |  |
| --- | --- | --- | --- | --- | --- | --- | --- | --- | --- | --- | --- | --- | --- | --- | --- | --- | --- | --- | --- |
| 0.88 (0.49,1.58) | DMF 240mgTID |  |  |  |  |  |  |  |  |  |  |  |  |  |  |  |  |  |  |
| 0.80 (0.44,1.44) | 0.90 (0.64,1.27) | GA20mg |  |  |  |  |  |  |  |  |  |  |  |  |  |  |  |  |  |
| 1.01 (0.22,4.75) | 1.14 (0.26,5.01) | 1.27 (0.29,5.60) | PON40mg |  |  |  |  |  |  |  |  |  |  |  |  |  |  |  |  |
| 0.76 (0.43,1.35) | 0.86 (0.66,1.13) | 0.96 (0.69,1.33) | 0.75 (0.17,3.29) | DMF 240mgBID |  |  |  |  |  |  |  |  |  |  |  |  |  |  |  |
| 0.69 (0.42,1.12) | 0.78 (0.54,1.11) | 0.86 (0.60,1.24) | 0.68 (0.16,2.98) | 0.90 (0.64,1.28) | FIN0.5mg |  |  |  |  |  |  |  |  |  |  |  |  |  |  |
| 0.61 (0.36,1.03) | **0.69 (0.53,0.89)** | 0.76 (0.56,1.03) | 0.60 (0.14,2.57) | 0.80 (0.63,1.01) | 0.88 (0.68,1.15) | PBO |  |  |  |  |  |  |  |  |  |  |  |  |  |
| 0.51 (0.01,26.97) | 0.57 (0.01,29.72) | 0.64 (0.01,33.05) | 0.50 (0.01,33.42) | 0.67 (0.01,34.43) | 0.74 (0.01,38.21) | 0.84 (0.02,42.94) | SIP0.25mg |  |  |  |  |  |  |  |  |  |  |  |  |
| **0.58 (0.36,0.94)** | **0.66 (0.45,0.95)** | 0.73 (0.49,1.07) | 0.57 (0.13,2.52) | 0.76 (0.53,1.09) | 0.84 (0.66,1.08) | 0.96 (0.72,1.26) | 1.14 (0.02,59.08) | FIN1.25mg |  |  |  |  |  |  |  |  |  |  |  |
| 0.55 (0.28,1.11) | 0.63 (0.36,1.10) | 0.70 (0.42,1.16) | 0.55 (0.12,2.57) | 0.73 (0.42,1.27) | 0.81 (0.49,1.33) | 0.91 (0.54,1.54) | 1.09 (0.02,57.98) | 0.96 (0.56,1.64) | FIN0.25mg |  |  |  |  |  |  |  |  |  |  |
| **0.53 (0.30,0.95)** | **0.60 (0.42,0.85)** | **0.67 (0.45,0.98)** | 0.53 (0.12,2.29) | **0.70 (0.50,0.98)** | 0.77 (0.54,1.11) | 0.88 (0.69,1.12) | 1.05 (0.02,54.00) | 0.92 (0.63,1.32) | 0.96 (0.54,1.70) | LAQ0.6mg |  |  |  |  |  |  |  |  |  |
| 0.44 (0.13,1.57) | 0.50 (0.16,1.63) | 0.56 (0.17,1.83) | 0.44 (0.07,2.81) | 0.58 (0.18,1.89) | 0.65 (0.20,2.10) | 0.73 (0.23,2.31) | 0.88 (0.01,52.94) | 0.77 (0.24,2.50) | 0.80 (0.23,2.83) | 0.84 (0.26,2.66) | LAQ0.3mg |  |  |  |  |  |  |  |  |
| 0.40 (0.11,1.45) | 0.45 (0.14,1.51) | 0.50 (0.15,1.69) | 0.40 (0.10,1.57) | 0.52 (0.16,1.74) | 0.58 (0.17,1.94) | 0.66 (0.20,2.14) | 0.79 (0.01,47.93) | 0.69 (0.21,2.31) | 0.72 (0.20,2.61) | 0.75 (0.23,2.50) | 0.90 (0.17,4.65) | PON20mg |  |  |  |  |  |  |  |
| **0.46 (0.22,0.95)** | **0.52 (0.29,0.92)** | 0.57 (0.32,1.04) | 0.45 (0.10,2.11) | 0.60 (0.34,1.05) | 0.66 (0.37,1.18) | 0.75 (0.45,1.26) | 0.90 (0.02,47.67) | 0.79 (0.44,1.41) | 0.82 (0.40,1.71) | 0.86 (0.49,1.52) | 1.03 (0.29,3.61) | 1.14 (0.32,4.13) | 2-CdA 3.5mg/kg |  |  |  |  |  |  |
| 0.38 (0.10,1.37) | 0.43 (0.13,1.43) | 0.47 (0.14,1.60) | 0.37 (0.09,1.48) | 0.50 (0.15,1.65) | 0.55 (0.16,1.84) | 0.62 (0.19,2.02) | 0.74 (0.01,45.26) | 0.65 (0.19,2.18) | 0.68 (0.19,2.47) | 0.71 (0.21,2.37) | 0.85 (0.16,4.39) | 0.94 (0.32,2.79) | 0.83 (0.23,2.99) | PON10mg |  |  |  |  |  |
| **0.42 (0.20,0.87)** | **0.48 (0.27,0.83)** | **0.53 (0.29,0.95)** | 0.42 (0.09,1.94) | **0.55 (0.32,0.96)** | 0.61 (0.35,1.08) | 0.69 (0.42,1.14) | 0.83 (0.02,43.80) | 0.73 (0.41,1.28) | 0.76 (0.37,1.56) | 0.79 (0.45,1.38) | 0.94 (0.27,3.30) | 1.05 (0.29,3.78) | 0.92 (0.58,1.47) | 1.11 (0.31,4.01) | 2-CdA 5.25mg/kg |  |  |  |  |
| 0.08 (0.00,1.79) | 0.09 (0.00,1.95) | 0.10 (0.00,2.17) | 0.08 (0.00,2.34) | 0.10 (0.00,2.26) | 0.12 (0.01,2.51) | 0.13 (0.01,2.81) | 0.16 (0.01,3.37) | 0.14 (0.01,2.98) | 0.14 (0.01,3.22) | 0.15 (0.01,3.25) | 0.18 (0.01,4.73) | 0.20 (0.01,5.32) | 0.17 (0.01,3.90) | 0.21 (0.01,5.63) | 0.19 (0.01,4.23) | SIP1.25mg |  |  |  |
| 0.07 (0.00,1.39) | 0.08 (0.00,1.52) | 0.08 (0.00,1.69) | 0.07 (0.00,1.84) | 0.09 (0.00,1.76) | 0.10 (0.00,1.95) | 0.11 (0.01,2.19) | 0.13 (0.01,2.62) | 0.12 (0.01,2.32) | 0.12 (0.01,2.50) | 0.13 (0.01,2.52) | 0.15 (0.01,3.69) | 0.17 (0.01,4.15) | 0.15 (0.01,3.04) | 0.18 (0.01,4.40) | 0.16 (0.01,3.29) | 0.84 (0.16,4.48) | SIP10mg |  |  |
| **0.05 (0.00,0.99)** | 0.06 (0.00,1.09) | 0.06 (0.00,1.21) | 0.05 (0.00,1.32) | 0.07 (0.00,1.26) | 0.07 (0.00,1.40) | 0.08 (0.00,1.57) | 0.10 (0.01,1.87) | 0.09 (0.00,1.66) | 0.09 (0.00,1.79) | 0.09 (0.00,1.81) | 0.11 (0.00,2.65) | 0.12 (0.01,2.98) | 0.11 (0.01,2.17) | 0.13 (0.01,3.16) | 0.12 (0.01,2.36) | 0.62 (0.13,3.10) | 0.75 (0.17,3.19) | SIP2mg |  |
| **0.02 (0.00,0.39)** | **0.02 (0.00,0.42)** | **0.03 (0.00,0.47)** | **0.02 (0.00,0.51)** | **0.03 (0.00,0.49)** | **0.03 (0.00,0.54)** | **0.03 (0.00,0.61)** | **0.04 (0.00,0.73)** | **0.04 (0.00,0.64)** | **0.04 (0.00,0.69)** | **0.04 (0.00,0.70)** | 0.05 (0.00,1.03) | 0.05 (0.00,1.16) | **0.05 (0.00,0.84)** | 0.05 (0.00,1.23) | **0.05 (0.00,0.91)** | 0.26 (0.06,1.13) | 0.31 (0.08,1.15) | 0.41 (0.12,1.40) | SIP0.5mg |

Note: Bold text indicates data with statistically significant differences.

**Supplementary Table 9:Network Inconsistency Test of T1**

| Side | Direct | | Indirect | | Difference | | P>z |
| --- | --- | --- | --- | --- | --- | --- | --- |
|  | Coef. | Std. Err. | Coef. | Std. Err. | Coef. | Std. Err. |  |
| DMF 120mgQD-DMF120mgTID | 0.2 | 1.06258 | -1.46 | 589.6232 | 1.66 | 589.624 | 0.998 |
| DMF 120mgTID-PBO | 0.2 | 0.9552014 | -1.46 | 606.6058 | 1.66 | 606.6066 | 0.998 |
| DMF 240mgTID-PBO | 0.9 | 1.090786 | -0.76 | 644.3319 | 1.66 | 644.333 | 0.998 |
| LAQ0.3mg- PBO | -0.4299996 | 1.029578 | 1.230041 | 625.0697 | -1.66 | 625.0719 | 0.998 |
| LAQ0.6mg-PBO | 0.8 | 1.26288 | 2.46 | 629.7754 | -1.66 | 629.7767 | 0.998 |

**Supplementary Table 10:Network Inconsistency Test of T2**

| Side | Direct | | Indirect | | Difference | | P>z |
| --- | --- | --- | --- | --- | --- | --- | --- |
|  | Coef. | Std. Err. | Coef. | Std. Err. | Coef. | Std. Err. |  |
| DMF 120mgQD-DMF 120mgTID | 0.3 | 0.9772153 | -0.0999996 | 560.5292 | 0.3999996 | 560.5297 | 0.999 |
| DMF 120mgQD-DMF240mgTID | -1.6 | 1.383621 | -2 | 648.6911 | 0.4 | 648.6924 | 0.999 |
| DMF 120mgQD -PBO | 0.4 | 1.238325 | 0.2 | 0.4021114 | 0.2 | 1.037933 | 0.847 |
| PON10mg-PBO | 0.1999955 | 1.917997 | 0.6000147 | 638.3449 | -0.4000193 | 638.3479 | 0.999 |
| PON20mg -PON40mg | 0.199993 | 286.8626 | 8.43775 | 2432312 | -8.237757 | 2432312 | 0.999 |
| PON20mg-PBO | 0.4 | 0.5455835 | 0.8 | 632.0478 | -0.4 | 632.0481 | 0.999 |
| PON40mg-PBO | 0.2 | 0.3297025 | 0.6 | 665.9865 | -0.4 | 665.9866 | 0.999 |

**Supplementary Table 11:Test for ring inconsistency in AE**

| Loop | IF | seIF | z_value | p_value | CI_95 | Loop_Heterog_tau2 |
| --- | --- | --- | --- | --- | --- | --- |
| DMF120mgTID-DMF240mgTID-PBO | 0.769 | 0.839 | 0.917 | 0.359 | (0.00,2.41) | 0 |
| DMF 120mgQD-DMF240mgTID-PBO | 0.769 | 0.727 | 1.058 | 0.29 | (0.00,2.19) | 0 |
| LAQ0.3mg-LAQ0.6mg-PBO | 0.741 | 0.541 | 1.371 | 0.17 | (0.00,1.80) | 0 |
| FIN0.5mg-PBO-GA20mg | 0.553 | 0.424 | 1.303 | 0.192 | (0.00,1.38) | 0 |
| FIN0.5mg-FIN1.25mg-PBO | 0.542 | 0.398 | 1.36 | 0.174 | (0.00,1.32) | 0 |
| FIN0.5mg-FIN1.25mg-IFN-β-1a | 0.541 | 0.412 | 1.313 | 0.189 | (0.00,1.35) | 0 |
| DMF240mgTID-PBO-GA20mg | 0.285 | 0.442 | 0.646 | 0.518 | (0.00,1.15) | 0 |
| DMF240mgTID-DMF240mgBID-PBO | 0.159 | 0.464 | 0.344 | 0.731 | (0.00,1.07) | 0 |
| DMF240mgBID-PBO-GA20mg | 0.127 | 0.445 | 0.285 | 0.776 | (0.00,1.00) | 0 |
| DMF240mgTID-DMF240mgBID-GA20mg | 0.07 | 0.51 | 0.137 | 0.891 | (0.00,1.07) | 0 |

**Supplementary Table 12:Network Inconsistency Test of AE**

| Side | Direct |  | Indirect |  | Difference |  |  |
| --- | --- | --- | --- | --- | --- | --- | --- |
|  | Coef. | Std. Err. | Coef. | Std. Err. | Coef. | Std. Err. | P>z |
| 2-CdA 3.5mg/kg-2-CdA 5.25mg/kg | 0.2218558 | 0.1767861 | 0.7170793 | 164.7741 | -0.4952235 | 164.7742 | 0.998 |
| 2-CdA 3.5mg/kg-PBO | -0.4188833 | 0.163357 | -0.1732718 | 83.13456 | -0.2456115 | 83.13472 | 0.998 |
| 2-CdA 5.25mg/kg-PBO | -0.640739 | 0.1675673 | -0.1416277 | 167.8644 | -0.4991113 | 167.8645 | 0.998 |
| DMF 120mgQD-DMF 240mgTID | 0.1177834 | 0.521991 | -1.270823 | 0.8712679 | 1.388606 | 1.029253 | 0.177 |
| DMF 120mgQD-PBO | -0.6908776 | 0.4606527 | 0.6977279 | 0.9694985 | -1.388605 | 1.029252 | 0.177 |
| DMF 120mgTID-DMF 240mgTID | -0.5402079 | 0.6001091 | -1.928814 | 0.9201981 | 1.388607 | 1.029253 | 0.177 |
| DMF 120mgTID-PBO | -1.348868 | 0.5475923 | 0.0397375 | 1.013698 | -1.388606 | 1.029252 | 0.177 |
| DMF 240mgTID-DMF 240mgBID | 0.3391503 | 0.2268559 | 0.0370803 | 0.5767178 | 0.30207 | 0.6220233 | 0.627 |
| DMF 240mgTID-PBO | -0.1638047 | 0.1908563 | -0.7139508 | 0.5816346 | 0.5501461 | 0.6103067 | 0.367 |
| DMF 240mgTID-GA20mg | -0.5823199 | 0.2506361 | -0.5557129 | 0.4041012 | -0.026607 | 0.480551 | 0.956 |
| DMF 240mgBID-PBO | -0.4456862 | 0.1898235 | -1.419882 | 0.6381109 | 0.9741959 | 0.6568831 | 0.138 |
| DMF 240mgBID-GA20mg | -0.9406615 | 0.2703614 | -0.7334325 | 0.3933954 | -0.207229 | 0.4874023 | 0.671 |
| FIN0.25mg-FIN0.5mg | 0.2300432 | 0.2446629 | 0.8238911 | 0.7436146 | -0.5938479 | 0.7919188 | 0.453 |
| FIN0.25mg-GA20mg | -0.0845775 | 0.2329712 | -0.6784273 | 0.7547951 | 0.5938498 | 0.7919188 | 0.453 |
| FIN0.5mg-FIN1.25mg | 0.2017615 | 0.1636222 | 0.7956091 | 0.7728201 | -0.5938476 | 0.7919189 | 0.453 |
| FIN0.5mg-IFN-β-1a | 0.5789146 | 0.2229038 | -0.3223486 | 0.618871 | 0.9012632 | 0.6409539 | 0.16 |
| FIN0.5mg-PBO | -0.3149972 | 0.2398863 | 0.2782302 | 0.2895326 | -0.5932274 | 0.3809555 | 0.119 |
| FIN0.5mg-GA20mg | -0.3146208 | 0.2478547 | -0.6115453 | 0.3087911 | 0.2969245 | 0.3959595 | 0.453 |
| FIN1.25mg-IFN-β-1a | 0.144075 | 0.2407094 | 1.045338 | 0.5985289 | -0.9012633 | 0.6409541 | 0.16 |
| FIN1.25mg-PBO | -0.2323235 | 0.232402 | -0.48222 | 0.3804863 | 0.2498964 | 0.4476785 | 0.577 |
| LAQ0.3mg-LAQ0.6mg | -0.4821253 | 0.3641107 | 1.000658 | 0.675563 | -1.482784 | 0.7343406 | 0.053 |
| LAQ0.3mg-PBO | -0.0290316 | 0.390951 | -1.511815 | 0.6289572 | 1.482784 | 0.7343407 | 0.053 |
| LAQ0.6mg-PBO | -0.250769 | 0.0804919 | -0.4084318 | 314.49 | 0.1576628 | 314.49 | 0.999 |
| PON10mg-PBO | -0.1341423 | 0.308897 | -0.7032404 | 640.2921 | 0.5690981 | 640.2924 | 0.999 |
| PON20mg-PBO | -0.1534178 | 0.3052775 | -0.7237158 | 650.0252 | 0.570298 | 650.0253 | 0.999 |
| PON40mg-PBO | 0.0224729 | 0.2949422 | -0.5468645 | 659.3981 | 0.5693374 | 659.3982 | 0.999 |
| SIP0.25mg-PBO | 0.3342768 | 0.4549509 | -1.172831 | 631.2828 | 1.507108 | 631.2833 | 0.998 |
| SIP0.5mg-PBO | -0.4122448 | 0.5453762 | -1.917114 | 624.0776 | 1.504869 | 624.0777 | 0.998 |
| SIP1.25mg-PBO | 0.6045672 | 0.4638398 | -0.9012642 | 634.6835 | 1.505831 | 634.6836 | 0.998 |
| SIP10mg-PBO | -1.77114 | 0.7903005 | -3.277454 | 632.8435 | 1.506314 | 632.8434 | 0.998 |
| SIP2mg-PBO | -2.464287 | 1.06046 | -3.970629 | 634.0243 | 1.506341 | 634.0237 | 0.998 |
| PBO-GA20mg | -0.5370212 | 0.2461168 | -0.1238074 | 0.2844138 | -0.4132138 | 0.3841455 | 0.282 |

**Supplementary Table 13:Test for ring inconsistency in SAE**

| Loop | IF | seIF | z_value | p_value | CI_95 | Loop_Heterog_tau2 |
| --- | --- | --- | --- | --- | --- | --- |
| LAQ0.3mg-LAQ0.6mg-PBO | 0.724 | 0.995 | 0.727 | 0.467 | (0.00,2.67) | 0 |
| FIN0.5mg-FIN1.25mg-IFN-β-1a | 0.401 | 0.411 | 0.976 | 0.329 | (0.00,1.21) | 0 |
| FIN0.5mg-FIN1.25mg-PBO | 0.4 | 0.327 | 1.225 | 0.22 | (0.00,1.04) | 0 |
| DMF240mgTID-DMF240mgBID-PBO | 0.191 | 0.432 | 0.442 | 0.659 | (0.00,1.04) | 0 |
| DMF240mgBID-PBO-GA20mg | 0.138 | 0.319 | 0.431 | 0.666 | (0.00,0.76) | 0 |
| DMF240mgTID-DMF240mgBID-GA20mg | 0.079 | 0.342 | 0.23 | 0.818 | (0.00,0.75) | 0 |
| FIN0.5mg-PBO-GA20mg | 0.056 | 0.395 | 0.141 | 0.888 | (0.00,0.83) | 0 |
| DMF240mgTID-PBO-GA20mg | 0.035 | 0.334 | 0.105 | 0.916 | (0.00,0.69) | 0 |

**Supplementary Table 14:Network Inconsistency Test of SAE**

| Side | Direct | | Indirect | | Difference | | P>z |
| --- | --- | --- | --- | --- | --- | --- | --- |
|  | Coef. | Std. Err. | Coef. | Std. Err. | Coef. | Std. Err. |  |
| 2-CdA3.5mg/kg-2-CdA5.25mg/kg | 0.0829564 | 0.2390134 | 0.6365591 | 180.8991 | -0.5536027 | 180.8993 | 0.998 |
| 2-CdA3.5mg/kg-PBO | -0.2837767 | 0.2617005 | -0.013291 | 86.37378 | -0.2704857 | 86.37418 | 0.998 |
| 2-CdA5.25mg/kg-PBO | -0.3667331 | 0.2549173 | 0.1840087 | 172.1996 | -0.5507419 | 172.1999 | 0.997 |
| DMF 240mgTID-DMF240mgBID | 0.1375692 | 0.1376586 | 0.5234258 | 0.8048913 | -0.3858566 | 0.8153787 | 0.636 |
| DMF 240mgTID-PBO | 0.3831323 | 0.1329696 | 0.232586 | 0.5630846 | 0.1505463 | 0.574403 | 0.793 |
| DMF 240mgTID-GA20mg | 0.1023857 | 0.2050717 | 0.1030916 | 0.3273756 | -0.0007059 | 0.3883412 | 0.999 |
| DMF 240mgBID-PBO | 0.2264701 | 0.1225909 | 0.3003497 | 0.7636442 | -0.0738797 | 0.7716199 | 0.924 |
| FIN0.25mg-FIN0.5mg | 0.0074092 | 0.1996288 | -0.1734553 | 0.3104387 | 0.1808645 | 0.368649 | 0.624 |
| FIN0.5mg-FIN1.25mg | -0.2040401 | 0.2781528 | -0.2779261 | 0.7072966 | 0.073886 | 0.7716214 | 0.924 |
| FIN0.5mg-GA20mg | -0.3758904 | 0.2958606 | -0.302005 | 0.6854016 | -0.0738854 | 0.7716215 | 0.924 |
| FIN0.5mg-IFN-β-1a | 0.1734231 | 0.1292025 | 0.0995373 | 0.7598258 | 0.0738859 | 0.7716214 | 0.924 |
| FIN0.5mg-PBO | -0.1997136 | 0.2798261 | -1.007473 | 0.5158771 | 0.8077593 | 0.5754866 | 0.16 |
| FIN1.25mg-IFN-β-1a | 0.0739505 | 0.1531348 | 0.323576 | 0.3003203 | -0.2496255 | 0.3375535 | 0.46 |
| FIN1.25mg-GA20mg | -0.1718503 | 0.3105068 | -0.1349074 | 0.228988 | -0.0369429 | 0.3858108 | 0.924 |
| FIN1.25mg-PBO | -0.6672138 | 0.2595225 | 0.1405473 | 0.5467919 | -0.8077611 | 0.5754867 | 0.16 |
| LAQ0.3mg-LAQ0.6mg | 0.0117865 | 0.150016 | -0.4830546 | 0.4132544 | 0.4948411 | 0.4393897 | 0.26 |
| LAQ0.3mg-PBO | -0.6129556 | 0.7441736 | 0.8350428 | 1.207397 | -1.447998 | 1.508702 | 0.337 |
| LAQ0.6mg-PBO | -0.0421105 | 0.6488925 | -1.490106 | 1.362351 | 1.447996 | 1.508701 | 0.337 |
| PON10mg-PON40mg | -0.1331876 | 0.1235387 | -0.3460433 | 308.114 | 0.2128556 | 308.1141 | 0.999 |
| PON10mg-PBO | -0.4749419 | 0.6011479 | -0.0925801 | 631.5574 | -0.3823618 | 631.5582 | 0.999 |
| PON20mg-PBO | -0.4172336 | 0.600686 | -0.0348208 | 636.7447 | -0.3824127 | 636.745 | 0.999 |
| PON40mg-PBO | 0.5108256 | 0.7420072 | 0.8930549 | 636.2568 | -0.3822293 | 636.2571 | 0.999 |
| SIP0.25mg-SIP0.5mg | -0.1774554 | 2.0089 | -0.3900981 | 631.9855 | 0.2126427 | 631.995 | 0.999 |
| SIP0.25mg-PBO | -3.382718 | 1.4704 | -3.595347 | 626.04 | 0.2126288 | 626.0447 | 0.999 |
| SIP0.5mg-PBO | 0.4713763 | 1.291856 | 1.212254 | 16355.94 | -0.7408774 | 16355.94 | 0.999 |
| SIP1.25mg-PBO | -2.027173 | 1.562354 | -2.239681 | 634.9441 | 0.212508 | 634.9486 | 0.999 |
| SIP10mg-PBO | -2.204218 | 1.524148 | -2.416842 | 631.5936 | 0.2126242 | 631.5981 | 0.999 |
| SIP2mg-PBO | -2.498549 | 1.503483 | -2.711192 | 622.1661 | 0.2126425 | 622.1707 | 0.999 |
| PBO-GA20mg | -0.3022485 | 0.1903337 | -0.2187008 | 0.2603887 | -0.0835477 | 0.3173091 | 0.792 |

**Supplementary Table 15:**Network Meta-analysis for ARR (Mean Difference, 95% CI)

| SIP2mg |  |  |  |  |  |  |  |  |  |  |  |  |  |  |  |  |  |  |  |
| --- | --- | --- | --- | --- | --- | --- | --- | --- | --- | --- | --- | --- | --- | --- | --- | --- | --- | --- | --- |
| -0.11 (-0.57,0.35) | PON40mg |  |  |  |  |  |  |  |  |  |  |  |  |  |  |  |  |  |  |
| -0.17 (-0.55,0.22) | -0.06 (-0.32,0.20) | FIN1.25mg |  |  |  |  |  |  |  |  |  |  |  |  |  |  |  |  |  |
| -0.17 (-0.55,0.21) | -0.06 (-0.33,0.20) | -0.00 (-0.03,0.03) | FIN0.5mg |  |  |  |  |  |  |  |  |  |  |  |  |  |  |  |  |
| -0.10 (-0.40,0.20) | 0.01 (-0.46,0.48) | 0.07 (-0.33,0.46) | 0.07 (-0.33,0.47) | SIP10mg |  |  |  |  |  |  |  |  |  |  |  |  |  |  |  |
| -0.19 (-0.57,0.19) | -0.08 (-0.35,0.18) | -0.02 (-0.09,0.05) | -0.02 (-0.09,0.05) | -0.09 (-0.49,0.31) | 2-CdA 3.5mg/kg |  |  |  |  |  |  |  |  |  |  |  |  |  |  |
| -0.17 (-0.65,0.32) | -0.06 (-0.46,0.34) | 0.00 (-0.30,0.31) | 0.01 (-0.30,0.31) | -0.07 (-0.56,0.43) | 0.02 (-0.28,0.33) | DMF 120mgQD |  |  |  |  |  |  |  |  |  |  |  |  |  |
| -0.19 (-0.58,0.19) | -0.09 (-0.35,0.18) | -0.03 (-0.09,0.04) | -0.02 (-0.09,0.04) | -0.09 (-0.49,0.30) | -0.00 (-0.08,0.07) | -0.03 (-0.33,0.28) | DMF 240mgBID |  |  |  |  |  |  |  |  |  |  |  |  |
| -0.19 (-0.66,0.28) | -0.08 (-0.32,0.15) | -0.02 (-0.30,0.26) | -0.02 (-0.29,0.26) | -0.09 (-0.57,0.39) | 0.00 (-0.28,0.28) | -0.02 (-0.43,0.39) | 0.01 (-0.27,0.29) | PON10mg |  |  |  |  |  |  |  |  |  |  |  |
| -0.20 (-0.58,0.19) | -0.09 (-0.35,0.17) | -0.03 (-0.10,0.04) | -0.03 (-0.09,0.04) | -0.10 (-0.50,0.30) | -0.01 (-0.08,0.07) | -0.03 (-0.33,0.27) | -0.00 (-0.05,0.04) | -0.01 (-0.29,0.27) | DMF 240mgTID |  |  |  |  |  |  |  |  |  |  |
| -0.20 (-0.58,0.18) | -0.09 (-0.36,0.17) | -0.03 (-0.10,0.04) | -0.03 (-0.10,0.04) | -0.10 (-0.50,0.30) | -0.01 (-0.05,0.03) | -0.03 (-0.34,0.27) | -0.01 (-0.08,0.07) | -0.01 (-0.29,0.27) | -0.00 (-0.08,0.07) | 2-CdA 5.25mg/kg |  |  |  |  |  |  |  |  |  |
| -0.24 (-0.63,0.15) | -0.13 (-0.40,0.14) | -0.07 (-0.15,0.01) | -0.07 (-0.14,0.01) | -0.14 (-0.54,0.26) | -0.05 (-0.15,0.05) | -0.07 (-0.39,0.24) | -0.05 (-0.14,0.04) | -0.05 (-0.34,0.23) | -0.04 (-0.13,0.05) | -0.04 (-0.14,0.06) | FIN0.25mg |  |  |  |  |  |  |  |  |
| -0.27 (-0.75,0.20) | -0.17 (-0.41,0.08) | -0.10 (-0.39,0.18) | -0.10 (-0.39,0.18) | -0.17 (-0.66,0.31) | -0.08 (-0.37,0.21) | -0.11 (-0.52,0.31) | -0.08 (-0.37,0.21) | -0.09 (-0.35,0.18) | -0.07 (-0.36,0.21) | -0.07 (-0.36,0.22) | -0.03 (-0.33,0.26) | PON20mg |  |  |  |  |  |  |  |
| -0.28 (-0.66,0.11) | -0.17 (-0.43,0.09) | **-0.11 (-0.17,-0.05)** | **-0.11 (-0.16,-0.05)** | -0.18 (-0.58,0.22) | **-0.09 (-0.16,-0.01)** | -0.11 (-0.42,0.19) | **-0.08 (-0.14,-0.03)** | -0.09 (-0.37,0.19) | **-0.08 (-0.13,-0.03)** | **-0.08 (-0.15,-0.00)** | -0.04 (-0.12,0.04) | -0.01 (-0.29,0.28) | GA20mg |  |  |  |  |  |  |
| -0.29 (-0.68,0.09) | -0.19 (-0.45,0.07) | **-0.13 (-0.19,-0.07)** | **-0.12 (-0.18,-0.06)** | -0.19 (-0.59,0.20) | **-0.10 (-0.17,-0.04)** | -0.13 (-0.44,0.18) | **-0.10 (-0.17,-0.03)** | -0.11 (-0.38,0.17) | **-0.10 (-0.17,-0.03)** | **-0.09 (-0.16,-0.03)** | -0.06 (-0.15,0.04) | -0.02 (-0.31,0.26) | -0.02 (-0.09,0.05) | LAQ0.6mg |  |  |  |  |  |
| **-0.41 (-0.82,-0.00)** | -0.30 (-0.85,0.24) | -0.24 (-0.73,0.24) | -0.24 (-0.72,0.24) | -0.31 (-0.73,0.11) | -0.22 (-0.70,0.26) | -0.24 (-0.81,0.32) | -0.22 (-0.70,0.27) | -0.22 (-0.78,0.33) | -0.21 (-0.70,0.27) | -0.21 (-0.69,0.27) | -0.17 (-0.66,0.32) | -0.14 (-0.70,0.42) | -0.13 (-0.62,0.35) | -0.12 (-0.60,0.37) | SIP0.5mg |  |  |  |  |
| -0.32 (-0.71,0.07) | -0.22 (-0.49,0.06) | **-0.15 (-0.24,-0.07)** | **-0.15 (-0.24,-0.06)** | -0.22 (-0.63,0.18) | **-0.13 (-0.24,-0.02)** | -0.16 (-0.47,0.16) | **-0.13 (-0.23,-0.02)** | -0.13 (-0.42,0.16) | **-0.12 (-0.23,-0.02)** | **-0.12 (-0.23,-0.01)** | -0.08 (-0.20,0.03) | -0.05 (-0.35,0.25) | -0.04 (-0.15,0.06) | -0.03 (-0.13,0.08) | 0.09 (-0.40,0.58) | IFN-β-1a |  |  |  |
| **-0.38 (-0.76,0.00)** | **-0.27 (-0.53,-0.02)** | **-0.21 (-0.26,-0.17)** | **-0.21 (-0.25,-0.17)** | -0.28 (-0.67,0.11) | **-0.19 (-0.24,-0.14)** | -0.21 (-0.52,0.09) | **-0.19 (-0.24,-0.13)** | -0.19 (-0.47,0.08) | **-0.18 (-0.24,-0.13)** | **-0.18 (-0.23,-0.13)** | **-0.14 (-0.22,-0.06)** | -0.11 (-0.39,0.17) | **-0.10 (-0.16,-0.05)** | **-0.09 (-0.13,-0.04)** | 0.03 (-0.45,0.51) | -0.06 (-0.16,0.04) | PBO |  |  |
| -0.53 (-1.06,0.01) | -0.42 (-0.87,0.03) | -0.36 (-0.73,0.02) | -0.35 (-0.73,0.02) | -0.43 (-0.97,0.12) | -0.34 (-0.71,0.04) | -0.36 (-0.76,0.04) | -0.33 (-0.70,0.04) | -0.34 (-0.80,0.12) | -0.33 (-0.70,0.04) | -0.33 (-0.70,0.05) | -0.29 (-0.67,0.09) | -0.25 (-0.72,0.21) | -0.25 (-0.62,0.13) | -0.23 (-0.61,0.14) | -0.12 (-0.72,0.49) | -0.20 (-0.59,0.18) | -0.15 (-0.52,0.23) | DMF 120mgTID |  |
| **-0.48 (-0.93,-0.02)** | **-0.37 (-0.73,-0.01)** | **-0.31 (-0.56,-0.06)** | **-0.31 (-0.56,-0.06)** | **-0.38 (-0.84,0.09)** | **-0.29 (-0.54,-0.03)** | -0.31 (-0.70,0.08) | **-0.28 (-0.54,-0.03)** | -0.29 (-0.66,0.08) | **-0.28 (-0.54,-0.03)** | -0.28 (-0.53,-0.02) | -0.24 (-0.50,0.02) | -0.21 (-0.58,0.17) | -0.20 (-0.46,0.05) | -0.18 (-0.43,0.06) | -0.07 (-0.61,0.47) | -0.16 (-0.42,0.11) | -0.10 (-0.35,0.15) | 0.05 (-0.40,0.49) | LAQ0.3mg |

Note: Bold text indicates data with statistically significant differences.

**Supplementary Table 16:** Network Meta-analysis for DAE (Odds ratio, 95% CI)

| FIN0.25mg |  |  |  |  |  |  |  |  |  |  |  |  |  |  |  |  |
| --- | --- | --- | --- | --- | --- | --- | --- | --- | --- | --- | --- | --- | --- | --- | --- | --- |
| 1.01 (0.47,2.19) | IFN-β-1a |  |  |  |  |  |  |  |  |  |  |  |  |  |  |  |
| 0.97 (0.18,5.24) | 0.95 (0.17,5.37) | LAQ0.3mg |  |  |  |  |  |  |  |  |  |  |  |  |  |  |
| 1.00 (0.08,12.15) | 0.99 (0.08,12.33) | 1.04 (0.06,19.03) | SIP0.25mg |  |  |  |  |  |  |  |  |  |  |  |  |  |
| 0.81 (0.22,3.03) | 0.80 (0.20,3.17) | 0.84 (0.11,6.25) | 0.81 (0.05,12.24) | DMF 120mgQD |  |  |  |  |  |  |  |  |  |  |  |  |
| 0.70 (0.42,1.17) | 0.69 (0.38,1.26) | 0.73 (0.14,3.75) | 0.70 (0.06,8.19) | 0.86 (0.25,3.04) | FIN0.5mg |  |  |  |  |  |  |  |  |  |  |  |
| 0.82 (0.07,10.00) | 0.81 (0.07,10.14) | 0.85 (0.05,15.65) | 0.82 (0.05,13.52) | 1.01 (0.07,15.34) | 1.17 (0.10,13.76) | SIP1.25mg |  |  |  |  |  |  |  |  |  |  |
| 0.59 (0.34,1.04) | 0.59 (0.30,1.14) | 0.61 (0.12,3.02) | 0.59 (0.05,6.70) | 0.73 (0.22,2.45) | 0.84 (0.58,1.24) | 0.72 (0.06,8.20) | PBO |  |  |  |  |  |  |  |  |  |
| 0.56 (0.16,1.92) | 0.56 (0.15,2.02) | 0.58 (0.08,4.08) | 0.56 (0.04,8.11) | 0.69 (0.21,2.30) | 0.80 (0.25,2.57) | 0.68 (0.05,9.92) | 0.95 (0.31,2.90) | DMF 120mgTID |  |  |  |  |  |  |  |  |
| **0.53 (0.33,0.87)** | 0.53 (0.27,1.05) | 0.55 (0.11,2.84) | 0.53 (0.05,6.20) | 0.66 (0.19,2.27) | 0.76 (0.52,1.12) | 0.65 (0.06,7.59) | 0.90 (0.62,1.30) | 0.95 (0.30,3.00) | GA20mg |  |  |  |  |  |  |  |
| **0.48 (0.27,0.87)** | **0.48 (0.23,0.96)** | 0.50 (0.10,2.52) | 0.48 (0.04,5.54) | 0.59 (0.18,1.98) | 0.68 (0.44,1.07) | 0.58 (0.05,6.77) | 0.81 (0.61,1.08) | 0.86 (0.28,2.62) | 0.90 (0.61,1.33) | DMF 240mgBID |  |  |  |  |  |  |
| **0.47 (0.26,0.85)** | **0.47 (0.23,0.95)** | 0.49 (0.10,2.48) | 0.47 (0.04,5.45) | 0.58 (0.18,1.89) | 0.67 (0.43,1.05) | 0.58 (0.05,6.68) | 0.80 (0.60,1.07) | 0.84 (0.29,2.49) | 0.89 (0.60,1.31) | 0.99 (0.74,1.31) | DMF 240mgTID |  |  |  |  |  |
| **0.38 (0.19,0.75)** | **0.38 (0.18,0.81)** | 0.39 (0.08,1.98) | 0.38 (0.03,4.43) | 0.47 (0.13,1.66) | **0.54 (0.32,0.93)** | 0.46 (0.04,5.42) | **0.64 (0.44,0.94)** | 0.68 (0.21,2.21) | 0.71 (0.42,1.20) | 0.79 (0.49,1.27) | 0.80 (0.50,1.29) | LAQ0.6mg |  |  |  |  |
| **0.34 (0.19,0.60)** | **0.33 (0.19,0.59)** | 0.35 (0.07,1.81) | 0.34 (0.03,3.94) | 0.41 (0.12,1.47) | **0.48 (0.35,0.67)** | 0.41 (0.03,4.82) | **0.57 (0.38,0.85)** | 0.60 (0.19,1.95) | **0.63 (0.40,0.99)** | 0.70 (0.44,1.12) | 0.71 (0.45,1.14) | 0.89 (0.51,1.53) | FIN1.25mg |  |  |  |
| **0.15 (0.03,0.91)** | **0.15 (0.02,0.93)** | 0.16 (0.02,1.61) | 0.15 (0.02,1.36) | 0.19 (0.02,1.50) | 0.22 (0.04,1.23) | 0.19 (0.02,1.66) | 0.26 (0.05,1.40) | 0.27 (0.04,2.06) | 0.29 (0.05,1.61) | 0.32 (0.06,1.76) | 0.32 (0.06,1.79) | 0.40 (0.07,2.27) | 0.45 (0.08,2.57) | SIP0.5mg |  |  |
| **0.14 (0.03,0.82)** | **0.14 (0.02,0.84)** | 0.15 (0.02,1.48) | 0.14 (0.02,1.24) | 0.18 (0.02,1.37) | 0.20 (0.04,1.11) | 0.17 (0.02,1.52) | 0.24 (0.05,1.26) | 0.26 (0.03,1.88) | 0.27 (0.05,1.46) | 0.30 (0.06,1.59) | 0.30 (0.06,1.62) | 0.38 (0.07,2.05) | 0.43 (0.08,2.32) | 0.94 (0.27,3.34) | SIP2mg |  |
| **0.08 (0.02,0.43)** | **0.08 (0.01,0.44)** | **0.08 (0.01,0.78)** | **0.08 (0.01,0.65)** | **0.10 (0.01,0.72)** | **0.11 (0.02,0.58)** | **0.10 (0.01,0.80)** | **0.14 (0.03,0.65)** | **0.14 (0.02,0.98)** | **0.15 (0.03,0.76)** | **0.17 (0.03,0.82)** | **0.17 (0.03,0.84)** | 0.21 (0.04,1.06) | 0.24 (0.05,1.20) | 0.53 (0.16,1.68) | 0.56 (0.19,1.68) | SIP10mg |

Note: Bold text indicates data with statistically significant differences.
